# Electron-induced deposition using Fe(CO)_4_MA and Fe(CO)_5_ – effect of MA ligand and process conditions

**DOI:** 10.3762/bjnano.15.45

**Published:** 2024-05-08

**Authors:** Hannah Boeckers, Atul Chaudhary, Petra Martinović, Amy V Walker, Lisa McElwee-White, Petra Swiderek

**Affiliations:** 1 Institute for Applied and Physical Chemistry (IAPC), Faculty 2 (Chemistry/Biology), University of Bremen, Leobener Str. 5, 28359 Bremen, Germany; 2 Department of Chemistry, University of Florida, Gainesville, Florida 32611, United States; 3 Department of Materials Science and Engineering RL10, University of Texas at Dallas, 800 W. Campbell Rd, Richardson, Texas 75080, United States

**Keywords:** autocatalytic growth, cryo-EBID, electron beam-induced deposition, heteroleptic iron precursor, thermal surface reactions

## Abstract

The electron-induced decomposition of Fe(CO)_4_MA (MA = methyl acrylate), which is a potential new precursor for focused electron beam-induced deposition (FEBID), was investigated by surface science experiments under UHV conditions. Auger electron spectroscopy was used to monitor deposit formation. The comparison between Fe(CO)_4_MA and Fe(CO)_5_ revealed the effect of the modified ligand architecture on the deposit formation in electron irradiation experiments that mimic FEBID and cryo-FEBID processes. Electron-stimulated desorption and post-irradiation thermal desorption spectrometry were used to obtain insight into the fate of the ligands upon electron irradiation. As a key finding, the deposits obtained from Fe(CO)_4_MA and Fe(CO)_5_ were surprisingly similar, and the relative amount of carbon in deposits prepared from Fe(CO)_4_MA was considerably less than the amount of carbon in the MA ligand. This demonstrates that electron irradiation efficiently cleaves the neutral MA ligand from the precursor. In addition to deposit formation by electron irradiation, the thermal decomposition of Fe(CO)_4_MA and Fe(CO)_5_ on an Fe seed layer prepared by EBID was compared. While Fe(CO)_5_ sustains autocatalytic growth of the deposit, the MA ligand hinders the thermal decomposition in the case of Fe(CO)_4_MA. The heteroleptic precursor Fe(CO)_4_MA, thus, offers the possibility to suppress contributions of thermal reactions, which can compromise control over the deposit shape and size in FEBID processes.

## Introduction

Focused electron beam-induced deposition (FEBID) is a state-of-the-art direct-write process for the fabrication of nanoscale materials and devices with arbitrary shape and size down to the sub-10 nm regime [1–3]. In FEBID, precursor molecules that contain the element of the desired material, typically a metal, are dosed via a gas inlet system onto a surface placed in a scanning electron microscope (SEM). There, the precursor is decomposed by the tightly focused electron beam to form a solid deposit. To provide the precursor with sufficient volatility, the metal atom to be deposited is surrounded by suitable ligands. In an ideal case, these ligands are converted to volatile species upon fragmentation of the precursor during electron irradiation and desorb from the surface while the desired element is deposited.

Owing to their magnetic properties, iron nanostructures produced by FEBID are of interest for diverse applications including magnetic data storage devices [4–6], tips for magnetic force microscopy [4,7], or sensors [4,8]. The same applies to cobalt nanostructures, which can be prepared with high purity and shape fidelity using, in particular, the precursor dicobalt octacarbonyl (Co_2_(CO)_8_) [4,6–8]. More recently, compounds containing more than one metallic element were developed as precursors for FEBID of bimetallic deposits [4,7–13]. Among those, HFeCo_3_(CO)_12_ has proven to yield deposits with high metal content and interesting magnetic properties [9,14–15]. However, considering that Fe is by orders of magnitude more abundant than Co, the further development of FEBID processes based on Fe precursors is appealing.

Although there were early attempts to use triiron dodecacarbonyl (Fe_3_(CO)_12_) or ferrocene (Fe(C_5_H_5_)_2_) for FEBID (see summary in [8]), iron pentacarbonyl (Fe(CO)_5_) has so far been the most prominent precursor for the deposition of iron, yielding metal contents up to 80 atom % without further purification [8]. Fe(CO)_5_ has also been used in FEBID processes that co-dosed dimethylgold(III) trifluoroacetylacetonate (Au(tfac)Me_2_) [16] or neopentasilane (Si_5_H_12_) [17] to produce Fe–Au alloy nanostructures and Fe–Si binary compounds, respectively. More recently, diiron nonacarbonyl (Fe_2_(CO)_9_) has received particular attention [5–6,18–20]. With this precursor and applying high beam energies, nanopillars with more than 90 atom % Fe were obtained [19], presumably related to electron beam heating effects. However, attempts to purify deposits with initial Fe contents of only 40 atom % by annealing up to 700 °C led to phase segregation into a highly pure and crystalline Fe phase and a carbonaceous material [5]. Notably, when the FEBID process is performed under ultrahigh vacuum (UHV) conditions instead of the usual high vacuum conditions prevalent in SEMs, deposits with purities up to 95 atom % Fe can be obtained from Fe(CO)_5_ [21]. Also, the well-controlled environment of such UHV studies revealed that autocatalytic decomposition of Fe(CO)_5_ contributes to the deposit growth in addition to the actual electron-induced fragmentation [22–25]. Although such autocatalytic deposit growth is favorable with respect to deposit purity as demonstrated in the framework of area-selective deposition initiated by electron beam-induced surface activation (EBISA) [24–25], it can compromise spatial control by the electron beam and selectivity when aiming for 3D nanostructures [3,26]. Strategies to suppress autocatalytic deposit growth are thus desirable to devise FEBID processes with optimum performance [27].

Autocatalytic growth (AG) of high-purity deposits leads to formation of individual crystallites as a consequence of precursor surface mobility at room temperature [21,24–25]. This mobility is suppressed when the surface is held at sufficiently low temperature. This was demonstrated by UHV experiments that performed FEBID from Fe(CO)_5_ at 200 K, which produced a continuous deposit [21]. More recently, such an approach was applied more extensively and introduced as cryo-FEBID [28–29]. For cryo-FEBID, the precursor is condensed at multilayer coverage onto a surface held at low temperature. The electron beam then writes patterns into the condensed layer followed by warming up to room temperature to remove the intact precursor from the non-irradiated areas. While cryo-FEBID is less versatile with respect to the fabrication of 3D nanostructures, the processing speed is much higher than for room-temperature FEBID, where the precursor is typically present at submonolayer coverage [29]. However, to the best of our knowledge, cryo-FEBID has not yet been applied to the fabrication of iron deposits.

Numerous fundamental studies have investigated the electron-induced fragmentation of isolated Fe(CO)_5_ in the gas phase [30–34], of clusters of the precursor [35–38], or of Fe(CO)_5_ adsorbed on surfaces [27,39–43] with the aim to provide insight into the chemical reactions inherent in the FEBID process. A recent surface science study was performed on Fe(CO)_5_ adsorbed on a Au surface held at 140 K with the precursor coverage amounting to a few molecular layers and irradiation performed at an electron energy of 500 eV [43]. The results revealed, in line with an earlier study [40], that the electron-driven decomposition of the precursor proceeds in two steps. First, electron irradiation removes on average 2.5 CO ligands from Fe(CO)_5_. This is followed by a second phase during which continued irradiation produces graphitic carbon and oxide material corresponding to about 20% of the initial CO ligands. However, these latter reactions do not further reduce the carbon and oxygen contents of the deposit. Throughout this conversion, all of the Fe content remains on the surface. Overall, electron irradiation of Fe(CO)_5_ multilayers at 140 K [43], thus, yields a deposit with significantly higher content of residual C and O than reported for room-temperature FEBID processes [8,21]. This indicates that thermal loss of further CO from partially decarbonylated intermediates Fe*_x_*(CO)*_y_* occurs at room temperature [26–27,43]. Notably and similar to Fe(CO)_5_, an average of three CO ligands is desorbed from the precursors Fe_2_(CO)_9_ and Fe_3_(CO)_12_ when irradiated at cryogenic temperature [43]. In these cases, though, the initial CO loss was more rapid, which was ascribed to the larger size of the molecules and consequently larger reactive scattering cross sections.

Overall, the results summarized above show that there is room for further improvements to devise FEBID processes for fabrication of iron nanostructures with optimum performance. Post-deposition purification approaches relying on O_2_ or H_2_O are not appropriate for iron deposits because they lead to oxidation [8]. The further improvement of iron deposition can, however, be tackled by rational design of the precursor molecules [44–47]. In the case of Fe precursors, the use of Fe_2_(CO)_9_ and Fe_3_(CO)_12_, for instance, is motivated by the idea that their higher inherent Fe content as compared to Fe(CO)_5_ should translate into a higher metal content of the deposits. However, this is not the case [43] unless conditions are applied that enable beam-induced heating of the deposit and, thus, enhance additional thermal reactions [19]. Furthermore, the somewhat better performance of Fe_2_(CO)_9_ [19] is obtained at the cost of a significantly lower volatility as compared to Fe(CO)_5_ [48].

**Figure 1:**
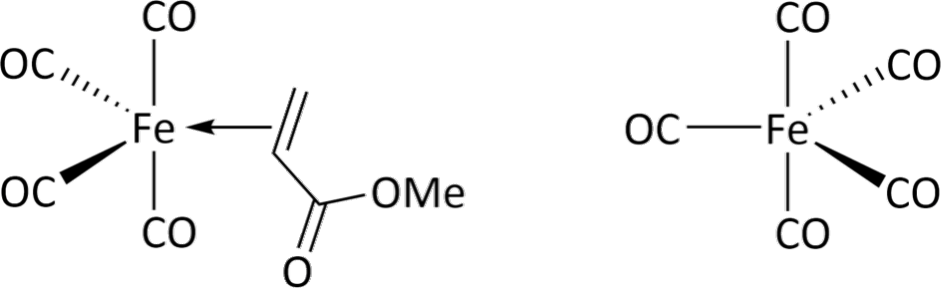
Molecular structures of (left) tetracarbonyliron(η^2^-methyl acrylate) (Fe(CO)_4_MA) and (right) iron pentacarbonyl (Fe(CO)_5_).

A strategy that has not been explored so far for the deposition of pure Fe is the use of heteroleptic Fe precursors, that is, compounds in which different types of ligands are attached to a single central Fe atom. In particular, hydrocarbons with C–C π bonds such as olefins bind to Fe via a bonding/backbonding scheme analogous to that seen in CO ligands. This involves electron donation from the olefin π bond to an empty d (or p) orbital of the metal. A filled d orbital backdonates its electrons into the empty π* orbital of the olefin. As neutral 2-electron donor species, olefins are expected to be labile ligands and, thus, to dissociate readily from the complex. This has, for instance, been shown for the case of Cu(I)(hfac)VTMS (hfac = hexafluoroacetylacetonate, VTMS = vinyltrimethylsilane, H_2_C=CH-Si(CH_3_)_3_) [3,49]. Therefore, we explore herein the electron-induced decomposition of a novel Fe precursor with an olefin ligand and a reduced number of CO ligands, namely, tetracarbonyliron(η^2^-methyl acrylate) (Fe(CO)_4_MA, MA = methyl acrylate, H_2_C=CH–COOCH_3_) (Figure 1). As a starting hypothesis, we anticipated that MA can readily desorb from the surface once it is dissociated from the precursor. Rapid desorption of MA would avoid electron-induced crosslinking reactions that have previously been observed for the closely related compound methyl methacrylate (MMA) [50]. Alternatively, the electron-induced fragmentation of MA may yield small and volatile products, including atomic H that, in turn, could potentially counteract oxidation of the deposit.

The present study uses a surface science approach to investigate the electron-induced decomposition of Fe(CO)_4_MA and the composition of the deposits resulting from experiments that mimic different FEBID processes. All experiments were repeated under the same conditions using Fe(CO)_5_ to elucidate the effect of the modified ligand architecture. The formation of volatile products formed by electron-induced fragmentation of the precursor adsorbed on a Ta surface held at 100 K was monitored by mass spectrometry. This was done either during electron irradiation in an electron-stimulated desorption (ESD) experiment or after irradiation when the surface temperature was increased during a thermal desorption spectrometry (TDS) experiment. ESD was monitored either by recording a mass spectrum or by following the time dependence of signals with specific *m*/*z* ratios to reveal the decomposition kinetics.

Iron deposits were produced from Fe(CO)_4_MA and Fe(CO)_5_ using an electron gun that irradiates the entire Ta surface. Therefore, we refer to our experiments as EBID and not as FEBID. Deposit compositions were monitored by Auger electron spectroscopy (AES). Using this approach, different processes were studied. (i) The EBID process was simulated by dosing a well-defined amount of the precursor onto the surface held at room temperature while performing electron irradiation. (ii) The cryo-EBID process was simulated by condensing the same amount of precursor onto the surface held at 100 K and subsequently performing electron irradiation of the condensed layer. (iii) In addition, AG was studied by dosing the precursor on a previous deposit produced by EBID from Fe(CO)_5_ and held at room temperature. Overall, the performance of Fe(CO)_4_MA in the different processes and in comparison to Fe(CO)_5_ is discussed.

## Results and Discussion

### Electron-stimulated desorption from condensed Fe(CO)_4_MA

The electron-induced fragmentation of Fe(CO)_4_MA was first investigated by ESD and post-irradiation TDS. The particular aim was to obtain insight into the fate of the MA ligand as result of electron irradiation of adsorbed Fe(CO)_4_MA. For reference, data for Fe(CO)_5_ are included in Figures S1–S3 of Supporting Information File 1.

**Figure 2:**
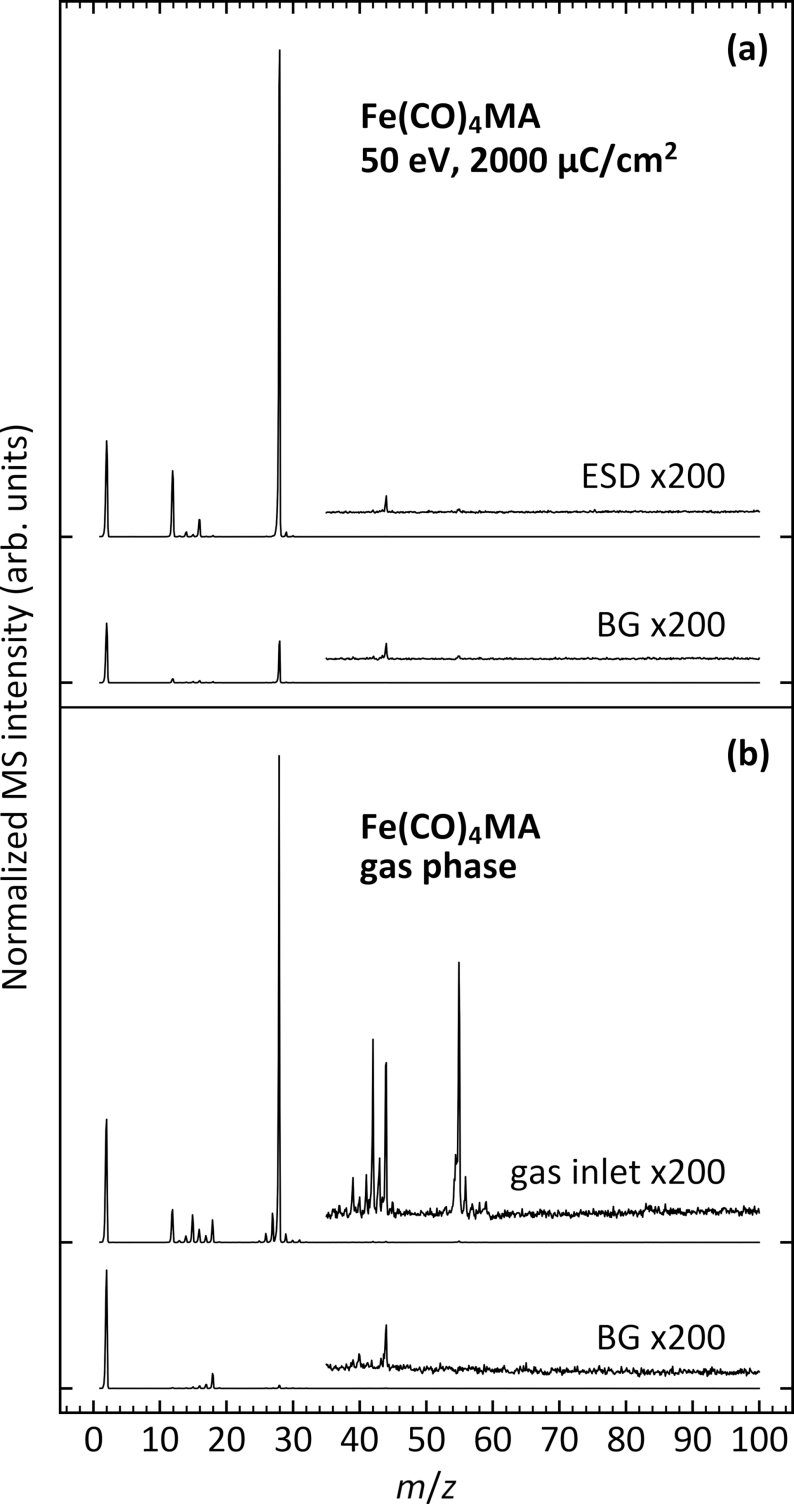
(a) ESD of neutral species during irradiation with 50 eV electrons from an Fe(CO)_4_MA multilayer on a Ta substrate held at 100 K (ESD). (b) Mass spectra recorded during leaking of Fe(CO)_4_MA into the UHV chamber (gas inlet). The thickness of the condensed layer was roughly five monolayers. Both data sets include a background mass spectrum (BG) recorded immediately prior to electron irradiation and precursor leakage, respectively. Ticks on the vertical axis indicate the baseline for each curve.

Figure 2a shows mass spectra acquired during ESD from a condensed multilayer of Fe(CO)_4_MA during irradiation with an energy of 50 eV. For comparison, the gas phase mass spectrum (MS) of Fe(CO)_4_MA, recorded during leaking of the precursor into the UHV chamber, is shown in Figure 2b. The latter is dominated by the *m*/*z* 28 signal (CO^•+^) which, together with smaller signals at *m*/*z* 12 (C^•+^) and *m*/*z* 16 (O^•+^), is ascribed to loss of the CO ligands. In addition, signals at *m*/*z* 55 (CH_2_CHCO^•+^), in the *m*/*z* 40–44 range, at *m*/*z* 27 (C_2_H_3_^•+^), and at *m*/*z* 15 (CH_3_^•+^) result from cleavage of the MA ligand. In particular, *m*/*z* 55 also appears as base peak in the MS of free MA [51]. A small *m*/*z* 56 signal can be assigned to Fe^+^ but is also present with an intensity of roughly 4% of the base peak in the MS of free MA [51]. In contrast, only CO is observed during ESD (Figure 2a) together with some H_2_ as obvious from the small increase of the *m*/*z* 2 signal. This result shows that the MA ligand does not desorb as a result of electron irradiation under cryogenic conditions. However, the minor release of H_2_ can be ascribed to C–H bond cleavage within the MA ligand or in potential products formed from MA during electron irradiation.

**Figure 3:**
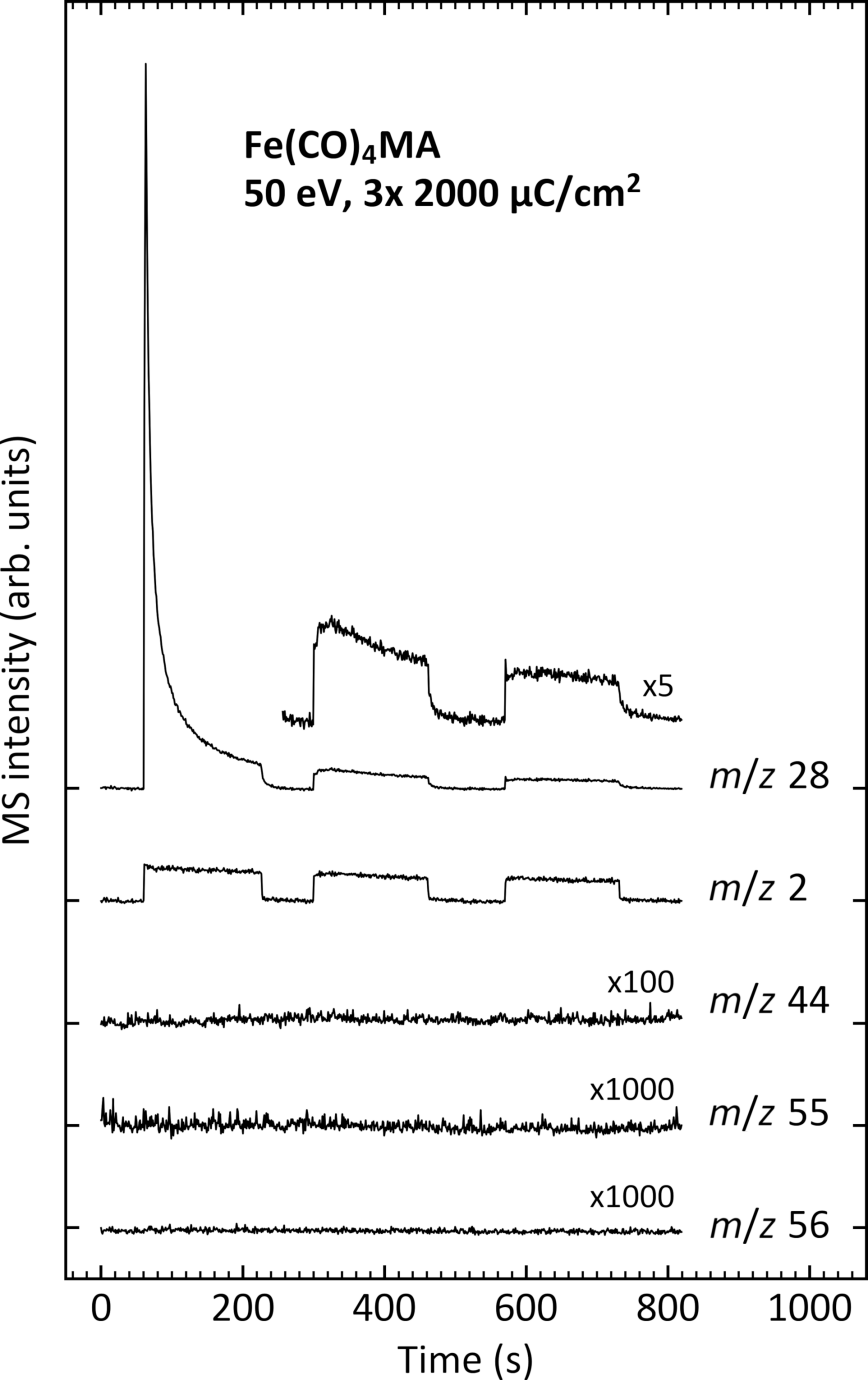
Time evolution of characteristic ESD signals recorded during irradiation with 50 eV electrons of an Fe(CO)_4_MA multilayer on a Ta substrate held at 100 K. The thickness of the condensed layer was roughly five monolayers. The signal at *m*/*z* 28 represents desorption of CO, *m*/*z* 2 is H_2_, and *m*/*z* 55 and 56 were recorded to reveal whether Fe-containing species or the free ligand or fragments thereof desorb. The signal at *m*/*z* 44 was monitored to observe the desorption of CO_2_. Irradiation was performed in three steps, each consisting of electron exposures of 2000 µC/cm^2^. Steps in the MS intensity mark the times when irradiation was switched on and off. Ticks on the vertical axis indicate the baseline level for each curve at the beginning of the experiment.

A second ESD experiment was performed under the same conditions to record the time evolution of specific *m*/*z* ratios during irradiation (Figure 3). Three irradiation steps of 2000 µC/cm^2^ each were interrupted by short periods without electron exposure. The start of each irradiation period is marked by a sudden increase of the *m*/*z* 28 (representative of CO) and *m*/*z* 2 (H_2_) signals, while both signals rapidly dropped in intensity when irradiation was switched off. Desorption of CO decays rapidly and exponentially during the first irradiation period, reflecting the depletion of the intact precursor molecules. However, a somewhat delayed CO desorption is observed at the start of the second irradiation. Here, the *m*/*z* 28 signal does not immediately decay after the sudden initial increase at the start of the irradiation period but continues to increase slowly during the first 30 s. We propose that at this later stage of irradiation the majority of CO that can be directly released into the gas phase by Fe–CO bond cleavage has been depleted. In this situation, desorption of CO via a two-step process becomes visible. In a first step, CO accumulates near the surface of the adsorbed precursor layer before it desorbs in the second step. CO can be delivered to the surface either by electron-induced fragmentation of the MA ligand or by diffusion of CO ligands cleaved from the precursor at deeper depths of the layer. As an alternative explanation, the delayed CO desorption might result from recombination of C and O that was released by electron-induced dissociation of CO ligands. This scenario, however, is less likely considering that such recombinative desorption of CO after thermal surface dissociation was only observed well above room temperature [52]. In contrast to the rapid loss of CO, desorption of H_2_ shows only a minor decay over all three periods. This points to a continuous C–H bond cleavage within the MA ligand or within products resulting from MA and supports that the MA ligand is decomposed at a slower rate as compared to the cleavage of CO from the complex. Again, the lack of *m*/*z* 55 and 56 in ESD shows that MA or the precursor itself do not desorb, while the negligible *m*/*z* 44 signal also rules out formation and immediate desorption of CO_2_ as an oxidation product of CO.

As an important finding, desorption of the intact MA ligand does not occur during electron irradiation under cryogenic conditions. Assuming that MA as a neutral ligand dissociates readily from the Fe(CO)_4_MA complex, the low temperature would prevent MA from desorbing. This explanation is supported by the previous finding that the closely related compound methyl methacrylate (MMA) desorbs around 170 K when adsorbed at multilayer coverages [50]. Free MA could also be dissociated by further electron irradiation. However, the absence of products other than CO and H_2_ in ESD (Figure 2a) implies that MA would be converted predominantly to products that are equally non-volatile at 100 K. We note that MMA has also been reported to polymerize under electron irradiation [50]. The same reactivity is anticipated for free MA, leading to larger oligomeric products with low volatility. Electron irradiation of such larger species can still lead to the release of H_2_ and possibly also of CO [53]. However, such polymerization reactions would be expected to lead to retention of a considerable amount of carbon in a deposit produced by electron irradiation of Fe(CO)_4_MA, which is not supported by the AES results described later.

**Figure 4:**
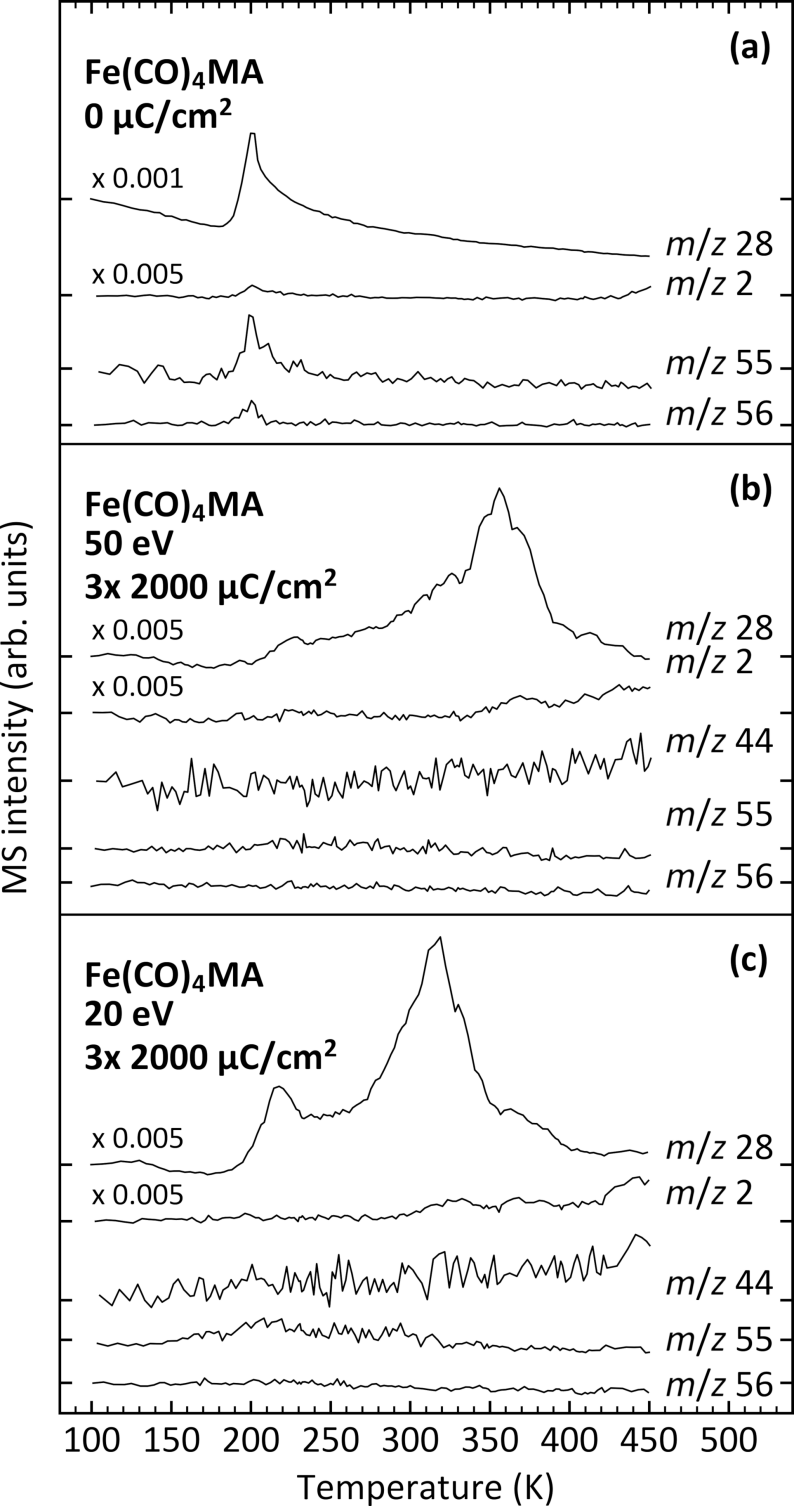
TDS obtained (a) from a pristine Fe(CO)_4_MA multilayer without electron irradiation (denoted as 0 µC/cm^2^), (b) from Fe(CO)_4_MA multilayers after three irradiation steps with a total electron exposure of 6000 µC/cm^2^ at 50 eV, and (c) after the same irradiation experiment performed at 20 eV. The thickness of the condensed layers prepared on a Ta substrate held at 100 K was roughly five monolayers. The slope of the baseline that is most noticeable in the *m*/*z* 28 TDS curve of the pristine Fe(CO)_4_MA multilayer is due to ongoing pump-down of gases present in the UHV chamber after leaking of the precursor. Ticks on the vertical axis indicate the baseline for each curve at the beginning of the experiment.

Post-irradiation TDS experiments can reveal the presence of products that become volatile when the sample temperature is increased and were performed to obtain further insight into the fate of the MA ligand (Figure 4). As reference, TDS data were first recorded at *m*/*z* 28, 2, 55, and 56 from a non-irradiated layer. They show the sharp desorption signal of intact Fe(CO)_4_MA near 200 K (Figure 4a). In contrast, the desorption signal of the intact precursor has disappeared after the 50 eV electron exposure experiment presented in Figure 3 (see Figure 4b) and also after an additional experiment performed with an electron energy of 20 eV (Figure 4c). ESD data recorded during electron irradiation at 20 eV are presented in Figure S4 of Supporting Information File 1. In both cases, a broad *m*/*z* 28 desorption signal has formed at higher temperature with its maximum around 350 K after electron irradiation at 50 eV and around 315 K after irradiation at 20 eV. The broad CO desorption signal indicates that further CO is lost from a deposit produced at 100 K as a consequence of thermal reactions when the temperature increases. The wide temperature range over which desorption occurs points to the formation of a variety of chemically different sites to which CO is bound [27]. The different shapes of the CO desorption signals obtained after irradiation at 50 and 20 eV furthermore point to structural differences between the deposits obtained at these two electron energies. This is very likely the result of a more extensive decomposition of the precursor at higher electron energy.

The TDS curves at *m*/*z* 55, which is the dominant MS signal of free MA [51], obtained after electron irradiation show a minor and, again, a broad desorption signal with onset near 150 K (Figure 4b,c). This is about 40 K below the onset in the TDS data obtained from the pristine Fe(CO)_4_MA layer (see also enlargement in Figure S5 of Supporting Information File 1). Considering the desorption temperature of 170 K reported for MMA [50], we ascribe the broad *m*/*z* 55 signal to the desorption of MA ligands released from Fe(CO)_4_MA during the prior electron irradiation. Notably, the signal is more visible after electron irradiation at 20 eV than at 50 eV which suggests that MA is more rapidly converted to other products at higher electron energy. This result indicates that at least part of the MA ligands can in fact be thermally removed from the deposit, which is also supported by the AES results described further on. We note, in addition, that the slight increase of the *m*/*z* 44 TDS signal may point to minor formation of CO_2_ as the oxidation product of CO via thermal reactions.

### Evaluation of deposit growth from AES data

**Figure 5:**
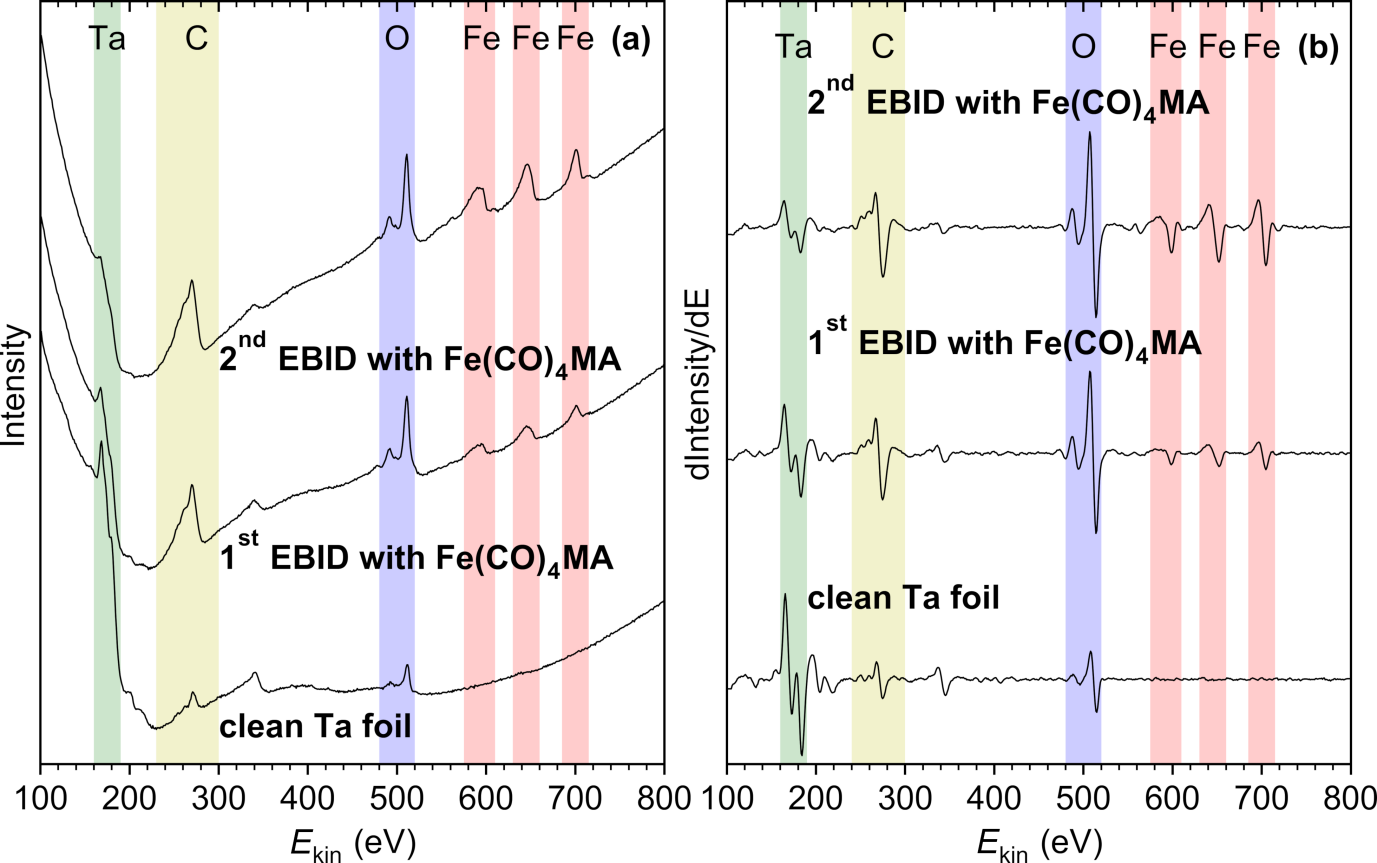
(a) AES recorded with an electron energy of 5 keV on a freshly sputtered Ta substrate (bottom). AES recorded on a deposit produced by dosing Fe(CO)_4_MA onto the Ta substrate held at room temperature during electron irradiation with an energy of 50 eV (middle). AES recorded after repetition of the EBID step on the first deposit (top). The electron dose was 10000 µC/cm^2^ in each EBID step. The amount of Fe(CO)_4_MA vapor used during the EBID steps would have produced roughly five monolayers of adsorbate on the substrate if held at 100 K. (b) Same AES data in differentiated form.

Deposits formed on the Ta substrate were characterized by recording Auger electron spectra (AES). Using the result of an EBID experiment as the example, we now discuss the information regarding deposit growth that can be deduced from the AES data. Figure 5a presents the raw data measured as a direct spectrum with intensity as function of electron energy, including an AES of the Ta foil after sputter cleaning as well as two subsequent EBID deposition steps. Figure 5b shows the derivative of the same raw data with respect to energy from which AES intensities were evaluated as peak-to-peak heights. The intensities were converted to a composition of each deposit (Fe/C/O) using the respective sensitivity factors [54]. The attenuation of the Ta_NNN_ signal gives an indication of growth of an overlayer on top of the substrate and can be converted to an overlayer thickness based on electron attenuation length (EAL) values [55]. The results of such analyses for all deposition experiments presented herein are summarized in Supporting Information File 1 (Tables S1 and S2). However, this approach to a quantitative analysis of AES data is only appropriate for overlayers with homogeneous thickness and distribution of the elements (referred to herein as scenario A) [54–55]. Therefore, we must first critically reflect upon the validity of composition and thickness data derived from the deposition experiments described herein.

According to earlier results [21,24–25], surface mobility of the precursor at room temperature in combination with contributions of AG likely leads to aggregation and, thus, to a non-homogeneous thickness. In the extreme case that each aggregate has a thickness that exceeds the escape depth of the Ta_NNN_ Auger electrons (referred to herein as scenario B), the attenuation of the Ta_NNN_ signal would rather reflect the decrease of uncovered Ta substrate area than an increase of the deposit thickness [27]. In contrast, a more homogeneous deposit is anticipated at cryogenic temperature, where surface mobility is lower [21]. As the situation in reality most likely lies somewhere between scenarios A and B, a deposit thickness cannot be reliably derived from the attenuation of the Ta_NNN_ signal. The attenuation of the Ta_NNN_ signal and the results summarized in Tables S1 and S2 of Supporting Information File 1 are thus merely used to qualitatively discuss deposit growth and to compare the different deposition processes performed herein and the two precursors Fe(CO)_4_MA and Fe(CO)_5_. In all experiments, the amount of precursor dosed onto the substrate was kept low enough to ensure that the Ta_NNN_ signal could still be quantified. For reference, we note that for a pure Fe deposit with homogeneous thickness (scenario A) and using the EAL in Fe (see section Experimental), the Ta_NNN_ signal would approach baseline levels for a deposit thickness of the order of 2 nm (Supporting Information File 1, Figure S6). This corresponds to roughly ten monolayers of Fe [56].

The increase of the Fe_LMM_ AES intensity can be used as an alternative measure of deposit growth [27]. In the case of scenario A, signals originating from an overlayer will show a saturation behavior as the thickness increases [55]. Here, it is important to note that the energy of Auger electrons and, thus, also the EAL increases in the sequence Ta_NNN_ (183 eV) < C_KLL_ (275 eV) < O_KLL_ (510 eV) < Fe_LMM_ (705 eV). In consequence, Fe_LMM_ Auger electrons can be detected from a larger depth than Ta_NNN_ electrons when travelling through the same material. For an Fe deposit with homogeneous thickness, this implies that the Fe_LMM_ signal will still increase above the thickness at which the Ta_NNN_ signal of the underlying substrate has disappeared. The probability that Fe_LMM_ Auger electrons escape from a pure Fe deposit in scenario A approaches zero for electrons originating from a depth of about 5 nm (Supporting Information File 1, Figure S6). The Fe_LMM_ signal, in consequence, would saturate when the overlayer thickness exceeds the same length. However, in scenario B, the intensity of the Fe_LMM_ signal would scale linearly with the surface area covered by such aggregates. Again, the actual situation may lie between these two extremes. Hence, we restrict our analysis to a qualitative discussion.

Regarding the analysis of the composition, we must consider a potentially inhomogeneous distribution of elements in the deposit. Prior to the actual deposition process, oxygen and carbon impurities are already present at the surface of the Ta substrate (Figure 5a,b, bottom). This is ascribed to surface reactions with residual gases such as CO and H_2_O (see below). After a deposition step, C_KLL_ and even more O_KLL_ electrons from these impurities at the interface between the Ta substrate and the deposit can contribute to the total signal within and even beyond the thickness regime where the Ta_NNN_ signal is still visible. This is a consequence of the larger EAL of C_KLL_ and O_KLL_ electrons as compared to that of Ta_NNN_ electrons. Furthermore, AG can contribute to the deposition in EBID after small Fe seeds have formed. This would lead to a higher deposit purity. For instance, a previous UHV study on EBID from Fe(CO)_5_ [27] revealed that the C_KLL_ signal first increased with the amount of precursor dosed during irradiation but then dropped again, while the Fe_LMM_ signal kept increasing. This indicated that the Fe content increased with increasing deposit thickness [27]. In the case of the thin layers deposited in the present experiment, the actual composition is, thus, also likely to vary from the first to the second deposition step. We recall, however, that in a situation near scenario A, AES probes the entire deposit. Therefore, again, trends between different deposition processes and precursors can be derived, but the effect of the different EALs and of potential inhomogeneity of the deposits must be carefully considered in the following discussion of the AES data.

### Thermal reactions of residual gases and of Fe(CO)_4_MA and Fe(CO)_5_ on the Ta substrate

**Figure 6:**
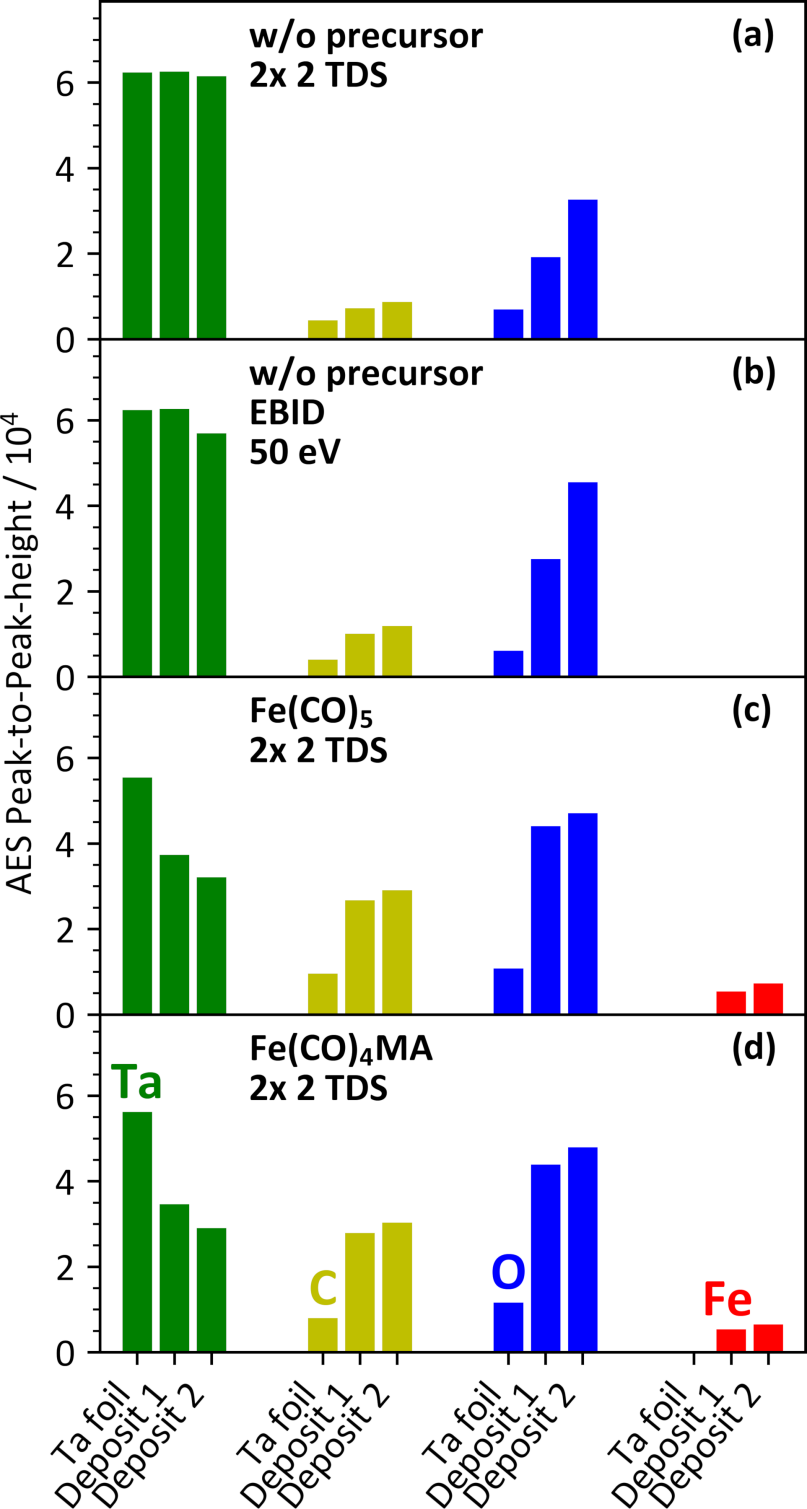
AES intensities for Ta_NNN_ (green), C_KLL_ (yellow), O_KLL_ (blue), and Fe_LMM_ (red) measured as peak-to-peak height within the differentiated AES data during four individual control experiments. In each experiment and for each element, the leftmost bar represents the AES data recorded after sputter cleaning of the Ta substrate. The other two bars represent data recorded after each of two subsequent process steps (deposits 1 and 2) during each of which (a) the substrate was cooled to 100 K followed by two TDS runs without prior leaking of precursor, (b) the substrate was irradiated with electrons (50 eV, 10000 µC/cm^2^) at room temperature without leaking of precursor, (c) the substrate was cooled to 100 K followed by two TDS runs performed each after leaking Fe(CO)_5_ (2.5 mTorr) onto the substrate held at 100 K, and (d) the substrate was cooled to 100 K followed by two TDS runs performed each after leaking Fe(CO)_4_MA (2.5 mTorr) onto the substrate held at 100 K. In each case, AES data were acquired when the substrate had returned to room temperature. The data in (c) and (d) each represent the average of two individual experiments (see also Supporting Information File 1, Tables S1 and S2).

Thermal reactions of precursors on the substrate can contribute to the first stages of deposit growth [27]. Also, residual gases may react with the Ta surface even under UHV conditions. As outlined above, elements that are, in consequence of such reactions, located at the interface between the Ta substrate and the deposit contribute to the AES intensities at the typical thickness of the deposits prepared herein. Therefore, we first performed a series of AES control experiments as summarized in Figure 6. This included the changes to the substrate by thermal reactions with residual gases (Figure 6a), by electron irradiation without precursor (Figure 6b), and by thermal reactions with both Fe(CO)_4_MA (Figure 6c) and Fe(CO)_5_ (Figure 6d). The results of these experiment serve as reference for the discussion of the actual deposition experiments (section Electron beam-induced deposition experiments with Fe(CO)_4_MA and Fe(CO)_5_).

Reactions with residual gases were enabled by cooling the substrate to 100 K followed by two subsequent TDS runs after which an AES was recorded. This sequence was performed two times. Figure 6a presents the AES data recorded from the Ta substrate after sputter cleaning and after the two TDS experiments. Small C_KLL_ and the O_KLL_ signals are already present in the first AES recorded after sputter cleaning of the substrate. They may have evolved during recording of the spectrum. However, we note that each AES within a particular experiment was measured from a different spot on the sample. Therefore, the continuous increase from one AES to the next must result from thermal reactions of the Ta surface with residual gases such as H_2_O and CO that are also seen in the BG MS data in Figure 2. The time between AES acquisitions within an experiment involving cryogenic temperatures was roughly 180 to 210 min, providing time for such reactions to occur. However, the attenuation of the Ta_NNN_ signal is negligible, pointing to a submonolayer coverage of carbon and oxygen.

Figure 6b represents an experiment performed with the substrate held at room temperature and irradiated twice with 50 eV electrons but without dosing a precursor. AES was again recorded from the Ta substrate after sputter cleaning and then after each electron irradiation step. The electron exposure was the same as also used in EBID and cryo-EBID experiments (see below). The time between two AES acquisitions was between 130 min and 160 min and, thus, somewhat shorter than in Figure 6a. In contrast, the O_KLL_ signal after electron irradiation (Figure 6b) is more intense than after thermal surface reaction (Figure 6a). This indicates that electron irradiation enhances the oxidation of the Ta surface by residual gases. However, the small attenuation of the Ta_NNN_ signal, despite being slightly more pronounced as compared to Figure 6a, is still typical of a coverage in the monolayer regime. For example, a carbon layer with homogeneous thickness of 0.2 nm (scenario A) would lead to an attenuation of the Ta_NNN_ signal by about 25% (Supporting Information File 1, Figure S6), which is more than seen in Figure 6b. We note again that the same attenuation would also result if 25% of the surface were covered with a few nanometers thick layer that fully screens the signal of the underlying substrate (scenario B). Scenario B, however, appears less likely because we anticipate that reactive species produced by electron exposure of residual H_2_O and CO would rather react with Ta than form a solid deposit by themselves. In fact, any carbonaceous deposit that might form as consequence of electron-induced dissociation of CO would also be etched by electron irradiation in presence of H_2_O [57–59].

Figure 6c and Figure 6d show the AES intensities from experiments performed in analogy to those presented by Figure 6a but this time with Fe(CO)_5_ or Fe(CO)_4_MA, respectively, condensed onto the Ta substrate before each TDS run. The total amount of precursor leaked before recording an AES was the same as the one applied in the EBID and cryo-EBID experiments (see below). After TDS, both precursors lead to similar AES intensities, namely, a small Fe_LMM_ signal and stronger C_KLL_ and O_KLL_ signals. The AES data recorded after the first two TDS runs with precursor (Figure 6c,d) show higher C_KLL_ and O_KLL_ intensities than the experiments without precursor (Figure 6a,b). In contrast, both signals as well as the Fe_LMM_ signal have hardly increased after the third and fourth TDS run. The data, thus, show a trend toward saturation. This also holds for the attenuation of the Ta_NNN_ signal, which reaches up to 50% in the case of Fe(CO)_4_MA (Figure 6d). Such an attenuation would indicate a surface coverage of roughly 0.3 nm of iron (one to two monolayers [56]) or 0.5 nm of carbon assuming a homogeneous thickness according to scenario A (Supporting Information File 1, Table S1, Table S2, and Figure S6) or a coverage of half of the Ta substrate by a thick deposit according to scenario B. The tendency towards saturation is in line with a self-limiting surface reaction in which the precursors are thermally dissociated on the Ta substrate (see also section Experimental) until the entire surface is covered by an overlayer with thickness defined by the dissociation products. This situation is in fact close to scenario A. Considering that the attenuation of the Ta_NNN_ signal by the deposit is at most 50% and that the EAL is larger for the signals originating from the deposit, AES must effectively probe the entire deposit here. In this situation, an estimate of the composition Fe/C/O may be derived from the average of the AES intensities over the two repetitions of the experiments as 1:7.8:5.4 for Fe(CO)_5_ and 1:9.0:6.2 for Fe(CO)_4_MA. The excess of carbon in the deposit as compared to the Fe/C/O composition of Fe(CO)_5_ (1:5:5) and Fe(CO)_4_MA (1:8:6) points again to incorporation of residual gases in the deposit. Overall, however, this result together with the TDS data presented in Figure S7 of Supporting Information File 1 indicates that most of the ligands remain bound to the surface or to precursor fragments following thermal surface reactions on the Ta substrate.

We note that scenario B would imply that thermal dissociation continues on top of the dissociated first layer of Fe(CO)_5_ or Fe(CO)_4_MA on the Ta substrate. Such AG has previously been revealed by AES when further Fe(CO)_5_ was dosed without electron irradiation onto a seed deposit produced by EBID and held at room temperature [27]. A control experiment in which the same amount of Fe(CO)_5_ was dosed directly onto the Ta substrate without EBID seed layer, however, led to much smaller Fe_LMM_ signals [27]. This difference already suggested that the Ta substrate does not initiate the thermal growth of a multilayer deposit from Fe(CO)_5_. The trend toward saturation seen in Figure 6c,d provides clear evidence that this is in fact the case. Dissociation products of both Fe(CO)_5_ and Fe(CO)_4_MA that are formed by thermal surface reactions on Ta are, thus, not capable of reacting with further precursor to sustain AG.

### Electron beam-induced deposition experiments with Fe(CO)_4_MA and Fe(CO)_5_

**Figure 7:**
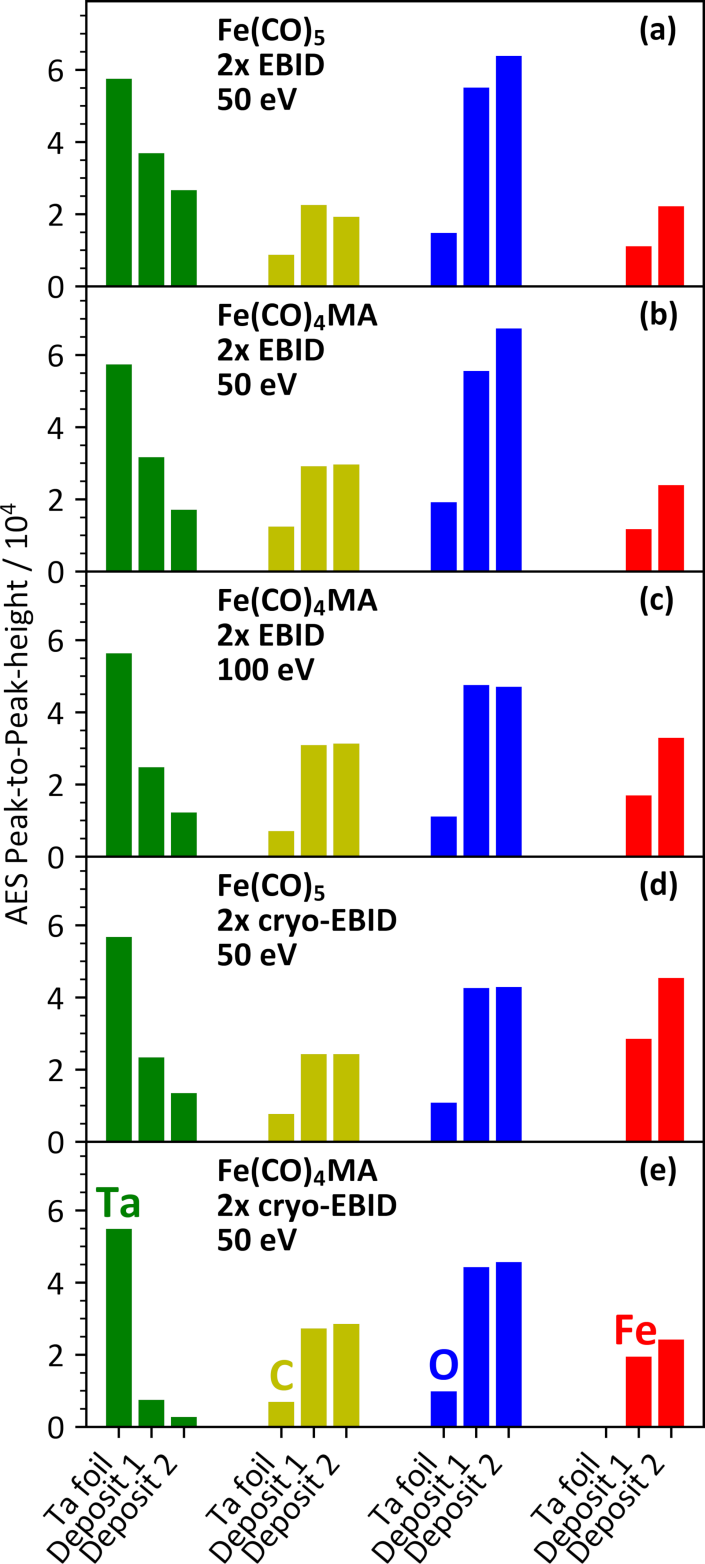
AES intensities for Ta_NNN_ (green), C_KLL_ (yellow), O_KLL_ (blue), and Fe_LMM_ (red) measured as peak-to-peak height within the differentiated AES data during five individual deposition experiments. In each experiment and for each element, the leftmost bar represents the AES data recorded after sputter cleaning of the Ta substrate. The other two bars represent data recorded after each of two subsequent deposition steps (deposits 1 and 2). EBID steps were performed with the substrate held at room temperature during (a) simultaneous irradiation with electrons (50 eV, 10000 µC/cm^2^) and leaking of Fe(CO)_5_ (5 mTorr), (b) simultaneous irradiation with electrons (50 eV, 10000 µC/cm^2^) and leaking of Fe(CO)_4_MA (5 mTorr), and (c) simultaneous irradiation with electrons (100 eV, 10000 µC/cm^2^) and leaking of Fe(CO)_4_MA (5 mTorr). For cryo-EBID steps with (d) Fe(CO)_5_ and (e) Fe(CO)_4_MA, the precursors (5 mTorr) were condensed onto the Ta substrate cooled to 100 K and irradiated with electrons (50 eV, 10000 µC/cm^2^) at that temperature. Each deposition step was followed by a TDS run. In each case, AES data were acquired when the substrate had returned to room temperature. All data represent the average of two individual experiments (see also Supporting Information File 1, Tables S1 and S2).

These deposition experiments aimed to evaluate the performance of Fe(CO)_4_MA in both EBID and cryo-EBID processes and as compared to the established precursor Fe(CO)_5_. Briefly, each experiment included two individual deposition steps that always used the same amount of precursor vapor. In EBID experiments, the Ta substrate was held at room temperature, and the precursor vapor was dosed onto the substrate during electron irradiation. In cryo-EBID experiments, the precursor was condensed on the Ta substrate held at 100 K, after which electron irradiation was performed. After each deposition step, the remaining volatile species were removed by a TDS run followed by annealing. AES was again measured after sputter cleaning the Ta substrate and after each deposition step. The AES intensities obtained in these experiments are summarized in Figure 7. The successful deposition of Fe by electron irradiation is evident from the higher intensity of the Fe_LMM_ signals as compared to the control experiments (Figure 6).

In all EBID experiments (Figure 7a–c), the Fe_LMM_ intensity increased by a factor of two from the first to the second deposition step, indicative of continued deposition controlled by the electron beam. In contrast, the C_KLL_ signal does not increase, and the O_KLL_ signal increases only slightly after the first EBID step. The AES intensities for Fe_LMM_ and O_KLL_ obtained after two EBID steps with an electron energy of 50 eV using Fe(CO)_5_ and Fe(CO)_4_MA agree within 10% (Figure 7a,b). In contrast, the C_KLL_ signal is about 50% more intense, and the attenuation of the Ta_NNN_ signal amounts to around 70% in the case of Fe(CO)_4_MA as compared to around 55% in the case of Fe(CO)_5_ (see Tables S1 and S2 of Supporting Information File 1). To rationalize the result, we consider again the two extreme cases of a deposit with homogeneous thickness (scenario A) and of the Ta substrate that is partially covered by thick aggregates (scenario B). Based on scenario A, a lower and upper limit for the thickness of the deposit can be derived from the attenuation of the Ta_NNN_ signal. This yields a thickness between 0.3 and 0.5 nm for Fe(CO)_5_ and between 0.45 and 0.80 nm for Fe(CO)_4_MA (Supporting Information File 1, Tables S1 and S2). Considering that the EAL for O_KLL_ is only about 25% shorter than for Fe_LMM_ electrons, both signals should still be far from saturation in this thickness regime. Therefore, the rapid saturation of the O_KLL_ signal would imply that less oxygen was co-deposited in the second EBID step than in the first, which can be ascribed to contributions of AG on the prior deposit (see also section Evaluation of deposit growth from AES data).

The same conclusion can also be derived within scenario B, where the decrease of the Ta_NNN_ signal relates to an increasing area covered by Fe-containing aggregates. We recall that aggregation is likely during a room-temperature EBID process [21,24–25]. However, if the increase in surface coverage reflected simply a larger number of aggregates or the growth of existing aggregates with constant composition, the O_KLL_ intensity should increase together with the Fe_LMM_ signal. Again, the saturation of the O_KLL_ signal would, thus, indicate that the material deposited in the second EBID step has a higher Fe content. It is difficult to rationalize this situation if the second EBID step would preferentially produce more aggregates. Such new aggregates would be formed on the same free Ta surface as those resulting from the first EBID step and, thus, should have the same composition. A more realistic explanation is that deposit growth during the second EBID step predominantly enlarges previous aggregates so that deposition involves contributions of AG, the latter enabling a higher Fe content.

While the actual deposit growth mode is most likely intermediate between scenarios A and B, both models lead to the conclusion that the composition of the deposit varies with ongoing deposition in the thickness regime investigated herein. Therefore, the composition derived from the intensity ratio Fe/C/O is only a crude estimate. Assuming, however, that the surface mobility and, thus, also the growth mode is similar for Fe(CO)_5_ and Fe(CO)_4_MA, we can at least qualitatively compare the composition after the second EBID step, which we state here as the average of the two individual experiments for each precursor. This estimate yields an Fe/C/O composition of 1:1.7:2.5 for Fe(CO)_5_ and of 1:2.4:2.4 for Fe(CO)_4_MA. The deviation of the composition between the two repetitions of the experiments is 30% for Fe(CO)_5_ and less than 10% for Fe(CO)_4_MA (see Tables S1 and S2 of Supporting Information File 1). The two compositions are surprisingly similar considering the higher amount of carbon in Fe(CO)_4_MA and suggests that the MA ligand is indeed efficiently removed in a room-temperature EBID process. For comparison, the EBID experiment performed at 100 eV using Fe(CO)_4_MA (Figure 7c) yields a deposit with higher Fe content and less oxygen (1:1.9:1.2). Also, the Ta_NNN_ signal is attenuated more strongly than at 50 eV (Figure 7b), indicative of a larger amount of deposited material. Increasing the electron energy, thus, leads to a more efficient deposition and a deposit with higher purity. We suggest that this relates to the larger number of secondary electrons released at higher electron energy.

Cryo-EBID experiments were performed with the same quantity of precursor as the EBID experiments (Figure 7d,e). In contrast to EBID, however, the Fe_LMM_ signals are more intense. This is, in particular, the case for Fe(CO)_5_, where the Fe_LMM_ signal after the second cryo-EBID step is about twice as large as after two EBID steps (Figure 7a), but also for the first cryo-EBID step with Fe(CO)_4_MA, which yields an Fe_LMM_ intensity that is about 70% higher than after the first EBID step (Figure 7b). Also, the Ta_NNN_ signal is more efficiently attenuated after the cryo-EBID steps as compared to EBID performed at 50 eV (Figure 7a,b). This indicates that more material was deposited than in the analogous EBID experiments. This was anticipated because a dense condensed precursor layer is irradiated in cryo-EBID, while EBID relies on a submonolayer equilibrium coverage established at room temperature [28–29]. However, the differences between Fe(CO)_5_ and Fe(CO)_4_MA are also more pronounced in the cryo-EBID experiments (Figure 7d,e). In line with a larger amount of deposited material, the Fe_LMM_ signals now also show a trend towards saturation, which is particularly obvious in the case of Fe(CO)_4_MA, where the Ta_NNN_ signal has nearly disappeared after the second cryo-EBID step (Figure 7e). The lower Fe_LMM_ intensity as compared to the cryo-EBID experiments with Fe(CO)_5_ (Figure 7d) is rationalized by a 30% higher intensity of the C_KLL_ signal, indicating that more carbon is co-deposited from Fe(CO)_4_MA.

Recalling that the surface mobility of the precursors in cryo-EBID performed at 100 K is reduced compared to the room-temperature EBID experiment [21] and also considering the strong attenuation of the Ta_NNN_ signal in cryo-EBID, we focus on scenario A as model for the evaluation of the resulting deposits. However, this ideal situation is, again, very likely not exactly fulfilled so that only trends can be derived from the analysis. Based on the assumption of a homogeneous overlayer, the attenuation of the Ta_NNN_ signal yields a thickness between 0.55 and 0.9 nm for Fe(CO)_5_ and between 1.8 and 1.9 nm for Fe(CO)_4_MA (Supporting Information File 1, Tables S1 and S2). At this thickness, the limited attenuation length of Fe_LMM_ Auger electrons becomes noticeable (Supporting Information File 1, Figure S6), which rationalizes the observed trend towards saturation. The average composition Fe/C/O deduced from the AES intensities amounts to 1:1.1:0.8 for Fe(CO)_5_ and 1:2.3:1.6 for Fe(CO)_4_MA. The difference in the carbon content between the two precursors is slightly more pronounced than in the EBID experiments (see above). However, the amount of carbon derived from the AES composition data is still considerably less than present in the precursors themselves. In particular, the composition Fe/C/O of Fe(CO)_4_MA is 1:8:6, which underlines that a large fraction of the MA ligands has been removed in the cryo-EBID process. Considering, however, the low volatility of the MA ligand at 100 K and the absence of signals of MA in ESD (see section Electron-stimulated desorption from condensed Fe(CO)_4_MA), it is likely that the ligand either desorbs during thermal annealing after electron irradiation or is decomposed to smaller and more volatile products during irradiation.

We note that removal of the MA ligand may be particularly favorable in the present cryo-EBID experiments as compared to an actual cryo-FEBID process where the thickness of the condensed precursor layers is typically in the hundreds of nanometers regime [28–29]. MA might be more easily trapped in such thick layers and, thus, be more prone to electron-induced polymerization as reported for condensed layers of MMA [50]. We also note that the somewhat higher amount of carbon and oxygen suggested by the compositions derived from the EBID experiments as compared to cryo-EBID may relate to thermal surface reactions (see section Thermal reactions of residual gases and of Fe(CO)_4_MA and Fe(CO)_5_ on the Ta substrate) within areas of the Ta substrate that are not covered by deposit aggregates. This underlines that the absolute figures obtained from the analysis of the present AES data should be merely used to discuss trends. In particular, the results should not be directly compared to results obtained by local elemental analyses performed in actual FEBID experiments.

### Autocatalytic deposit growth from Fe(CO)_4_MA and Fe(CO)_5_

**Figure 8:**
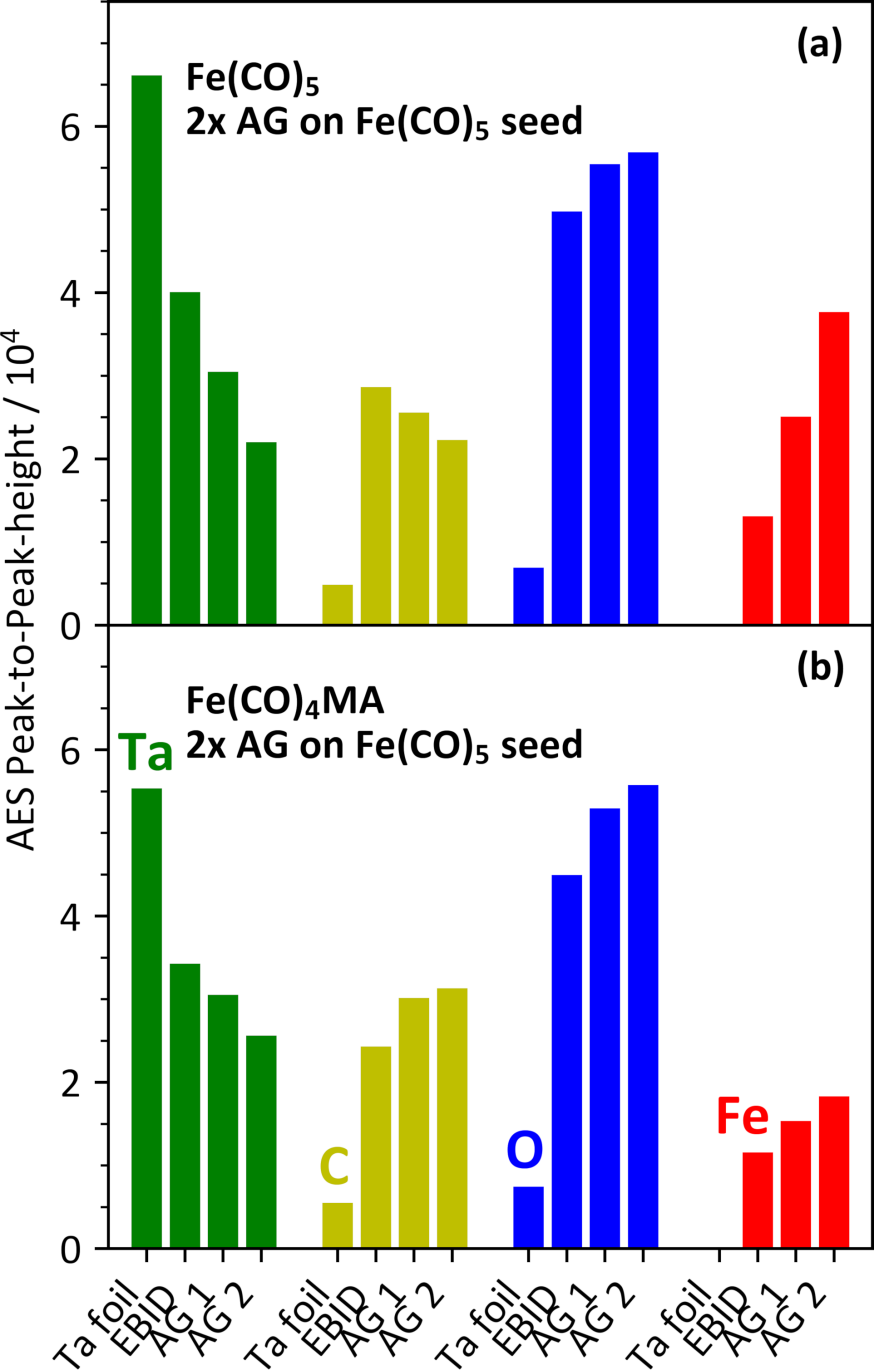
AES intensities for Ta_NNN_ (green), C_KLL_ (yellow), O_KLL_ (blue), and Fe_LMM_ (red) measured as peak-to-peak height within the differentiated AES data during two individual deposition experiments. In each experiment and for each element, the leftmost bar represents the AES data recorded after sputter cleaning of the Ta substrate. The second bar represents the AES data recorded after deposition of an Fe seed layer from Fe(CO)_5_ by EBID which was performed during simultaneous irradiation with electrons (50 eV, 10000 µC/cm^2^) and leaking of Fe(CO)_5_ (5 mTorr) with the substrate held at room temperature. The other two bars represent data recorded after each of two subsequent AG steps (AG 1 and 2) using (a) Fe(CO)_5_ and (b) Fe(CO)_4_MA. AG was performed by leaking the precursors (5 mTorr) onto the substrate held again at room temperature. Each deposition step was followed by a TDS run. In each case, AES data were acquired when the substrate had returned to room temperature. All data represent the average of two individual experiments (see also Supporting Information File 1, Tables S1 and S2).

The ability of Fe(CO)_4_MA to sustain AG was evaluated according to an approach described previously [27]. A sequence of deposition steps was performed in which first an Fe seed layer was produced by EBID from Fe(CO)_5_ using the same conditions as in one single EBID step of Figure 7a. This was followed by two steps of dosing either Fe(CO)_5_ or Fe(CO)_4_MA onto the seed deposit. AES was recorded on the sputter-cleaned Ta substrate and after each deposition step. The resulting AES intensities are summarized in Figure 8. The data show a continuous increase of the Fe_LMM_ signals with increasing number of deposition steps. The noticeable AG of the deposit from Fe(CO)_5_ (Figure 8a) is in line with the previous result [27]. However, a much smaller intensity increase and also a less pronounced attenuation of the Ta_NNN_ signal was observed when AG was performed with Fe(CO)_5_MA (Figure 8b), pointing to a lesser extent of thermal surface reactivity on the Fe seed deposit in this case. We, thus, conclude that the MA ligand inhibits the thermal decomposition of Fe(CO)_5_MA. As discussed earlier, the thermal decomposition of Fe(CO)_5_ on a growing Fe deposit is a reaction in which leaving CO ligands and the remaining Fe(CO)*_x_* fragment attach to the underlying Fe surface [52,60]. While the initial surface decomposition must be driven by the energy gain upon binding of the CO ligands and the Fe(CO)*_x_* fragment to the Fe surface, AG is sustained at temperatures that are high enough to enable desorption of CO [52,60]. We propose that these reactions are energetically less favorable for the MA ligand. This is also supported by the Fe/C/O compositions obtained after the individual deposition steps (Supporting Information File 1, Tables S1 and S2). While the initial Fe seed deposit exhibits an Fe/C/O ratio of about 1:4:3 in all individual experiments, it evolved to about 1:2:2 after the first AG step and to 1:1:1 after the second AG step when AG was performed with Fe(CO)_5_, indicating an increase of the Fe content with ongoing deposit growth. In contrast, a final Fe/C/O ratio of about 1:2:3 was obtained after two AG steps using Fe(CO)_4_MA.

**Figure 9:**
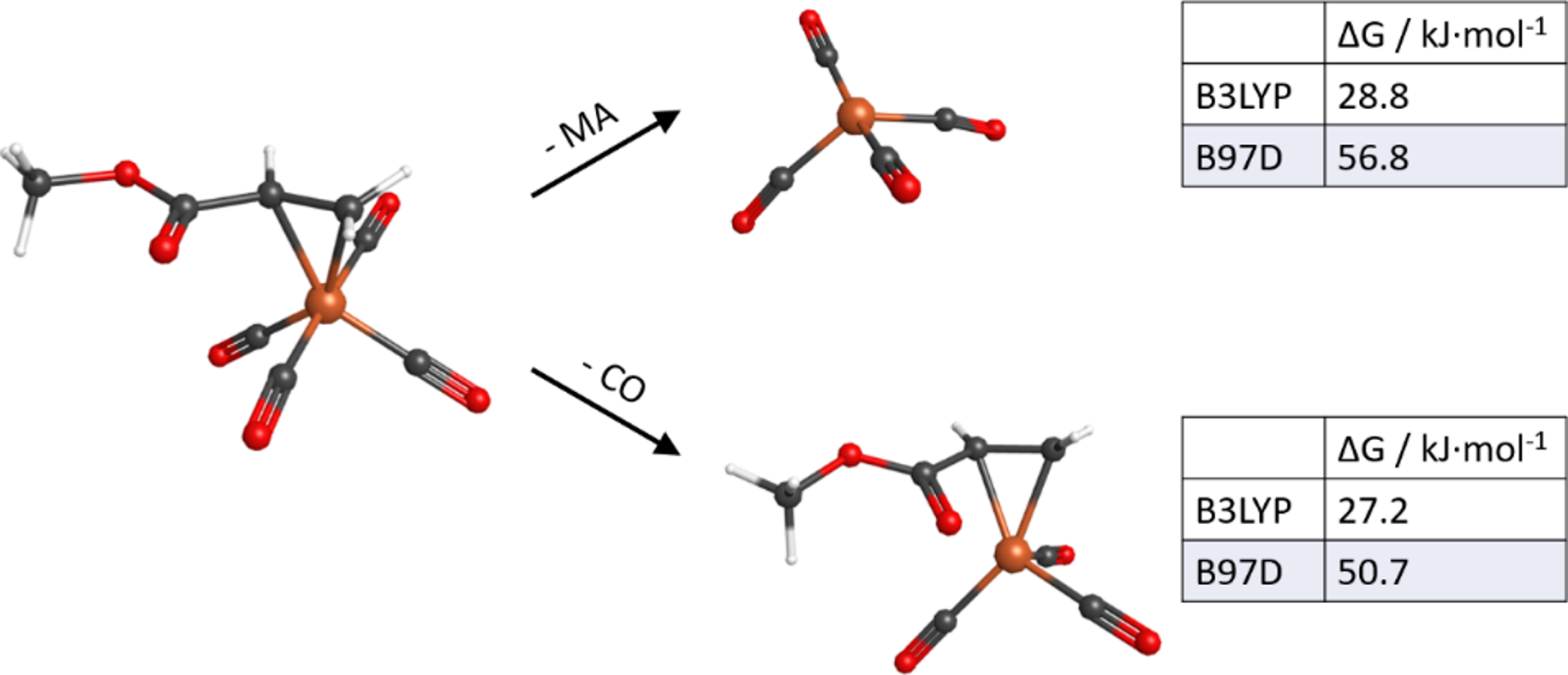
Free Gibbs energy for thermal loss of the MA ligand (top) and the CO ligand (bottom) from Fe(CO)_4_MA obtained from B3LYP and B97D calculations with LANL2DZ basis set for Fe and 6-311+G(2d,p) basis set for other elements.

The higher carbon and oxygen contents of the deposits obtained by thermal decomposition of Fe(CO)_4_MA most likely relate to the persistence of a part of the MA ligands. This is also supported by calculated free reaction enthalpies for the loss of either the MA ligand or the CO ligand from gas phase Fe(CO)_4_MA (Figure 9), which are in both cases lower than 30 kJ/mol using B3LYP and lower than 60 kJ/mol as obtained from the B97D dispersion corrected functional. The low value for MA indicates that this ligand is only weakly bound in Fe(CO)_4_MA. Also, the calculated value for loss of CO is significantly lower than the energy of around 176 kJ/mol required to dissociate the first CO from Fe(CO)_5_ [61]. We propose that the energy required for loss of a CO ligand from Fe(CO)_4_MA is counterbalanced by MA changing its coordination mode when CO is expelled. This change of coordination is also seen from the close distance of the carbonyl oxygen of MA to the Fe center in the Fe(CO)_3_MA product (see Figure 9, bottom). Thereby, the MA ligand becomes more tightly bound. A related change in the coordination mode of MA from η^2^ to η^4^ has been previously reported to occur upon photochemical CO loss from Fe(CO)_4_MA [62–63]. While a more extensive theoretical surface study is beyond the scope of the present work, we hypothesize that, in a thermal reaction, the MA ligand deactivates the surface of the growing deposit to some extent. Fe(CO)_4_MA is therefore most likely a suitable precursor when the aim is to suppress unwanted AG.

## Conclusion

The electron-induced decomposition of the heteroleptic potential FEBID precursor Fe(CO)_4_MA was investigated by surface science experiments under UHV conditions. The comparison with Fe(CO)_5_ revealed the effect of the modified ligand architecture on the deposit formation.

ESD experiments showed that CO and H_2_ desorb from Fe(CO)_4_MA upon electron irradiation under cryogenic conditions, and only small amounts of the intact MA ligand were observed in post-irradiation TDS. In contrast, the deposits prepared by EBID from the two precursors were surprisingly similar. This demonstrates that electron irradiation efficiently cleaves the neutral MA ligand from the precursor. Deposits prepared by cryo-EBID from Fe(CO)_4_MA exhibited about twice as much carbon and oxygen as compared to cryo-EBID from Fe(CO)_5_. In general, however, the relative amount of carbon in deposits prepared from Fe(CO)_4_MA was considerably less than the amount of carbon in the MA ligand. This result also indicates that electron-induced polymerization of the MA ligands does not play a major role in deposit formation. In contrast, the low carbon content and a delayed desorption of CO in later stages of ESD suggest that the MA ligand is efficiently fragmented during deposit formation at least in cryo-EBID. Desorption during room-temperature EBID could not be monitored because of the excess of intact precursor in the vacuum chamber during dosing. In the absence of electron-induced polymerization, however, thermal desorption of MA after electron-induced dissociation from Fe(CO)_4_MA is a conceivable pathway for the removal of carbon from the deposit.

In addition to deposit formation by electron irradiation, the thermal decomposition of Fe(CO)_4_MA and Fe(CO)_5_ on an Fe seed layer prepared by EBID was compared. While Fe(CO)_5_ clearly sustained the continued AG of the deposit, considerably less Fe was thermally deposited from Fe(CO)_4_MA. Therefore, we conclude that the MA ligand inhibits the thermal decomposition of the precursor. The heteroleptic precursor Fe(CO)_4_MA, thus, offers the possibility to suppress contributions of AG, which can compromise control over the deposit shape in a room-temperature FEBID process. Heteroleptic precursors such as Fe(CO)_4_MA are, thus, promising candidates for FEBID of iron. Alternative ligands will be considered in the future to further optimize the processes for the deposition of iron.

## Experimental

### Precursors

**General.** Glassware was flame-dried or oven-dried before use. Solvents (i.e., benzene and hexane) were purified using an MBraun MB-SP solvent purification system and stored over 4 Å molecular sieves before use. Reagents were purchased from Sigma-Aldrich and used without further purification. Deuterated solvent (benzene-*d*_6_) for NMR was purchased from Cambridge Isotopes Lab and was stored over 4 Å molecular sieves for 24 h prior to use. IR spectra in hexane were obtained on a PerkinElmer Spectrum ONE FT-IR spectrometer using a solution cell equipped with NaCl windows and a path length of 1.0 mm.

**Fe(CO)_5_.** Fe(CO)_5_ was purchased from Sigma-Aldrich in a stated purity of >99.99%.

**Fe(CO)_4_MA.** Fe(CO)_4_MA was synthesized in Gainesville using a modification of the reported literature procedure [64]. In a nitrogen-filled glovebox, a solution of Fe_2_(CO)_9_ (1.0 g, 2.7 mmol) in 20 mL of benzene was prepared in a 100 mL Schlenk flask. To this solution, methyl acrylate (0.24 mL, 2.7 mmol) was added. The reaction mixture was stirred for 4 h at 45 °C, and then the solvent was removed under vacuum using Schlenk techniques. The crude product was then sublimed at room temperature at 700 mTorr overnight, which afforded the yellowish product Fe(CO)_4_MA (300.0 mg, 43%) as supported by NMR and IR analyses (Figures S8 and S9, Supporting Information File 1), with comparison to literature data [64].

### UHV setup

All experiments were performed in Bremen in an ultrahigh vacuum (UHV) setup with a base pressure of about 10^−10^ mbar using a polycrystalline Ta sheet as a substrate for adsorption of precursors and deposition processes. The setup consists of two chambers described previously [27,57–58,65] between which the Ta sheet can be translated. The lower chamber is equipped with a commercial flood gun (SPECS FG 15/40) for electron irradiation of the entire Ta substrate and a quadrupole mass spectrometer (QMS) residual gas analyzer (Stanford, 300 amu) that produces ions by electron ionization with an energy of 70 eV. The upper chamber contains an Auger electron spectrometer (AES) (STAIB DESA 100) and a sputter gun operated with Ar^+^ ions at an energy of 3 keV. Prior to each experiment, the substrate was sputter-cleaned for 30–60 min to remove any previous deposit as deduced from the lack of Fe_LMM_ AES signals. The Ta sheet is attached to a liquid nitrogen bath cryostat for cooling to about 100 K. Resistive heating is provided by two thin Ta ribbons spot-welded to the thicker Ta sheet. Immediately before dosing of the precursor, adsorbed residual gases were, thus, removed by annealing to 450 K. The total irradiated area of the substrate is 5 cm^2^.

### Preparation of adsorbed precursor layers

After shipping to Bremen, Fe(CO)_4_MA was transferred to a reservoir under N_2_ atmosphere. Both precursors were degassed by repeated freeze-pump-thaw cycles. The Fe(CO)_4_MA reservoir was cooled using liquid nitrogen between gas inlets and stored in a freezer when it was not used for preparation. Vapors of the precursors were leaked into the UHV chamber via a stainless steel tube with an opening pointing toward the Ta sheet held at 100 K. The resulting effusive molecular beam is sufficiently divergent to allow for condensation across the entire Ta substrate. The amount of dosed vapor was defined by the pressure drop in the gas manifold upon leaking as measured in units of millitorrs with a capacitance manometer. TDS data of the resulting adsorbates were recorded by heating the substrate at a constant rate of 1 K/s while monitoring characteristic *m*/*z* ratios using the QMS. A TDS run was terminated by annealing to 450 K for 30 s. According to a previous estimate, a pressure drop of roughly 1 mTorr produced a monolayer coverage of Fe(CO)_5_ [27]. An estimate for Fe(CO)_4_MA was obtained herein by leaking varying amounts of vapor onto the substrate and recording TDS. While a desorption signal in the *m*/*z* 28 TDS curves already appears well below a gas dose of 1 mTorr and develops into a sharp peak near 200 K above that dose, signals in the *m*/*z* 55 and 56 curves, indicative of the desorption of Fe(CO)_4_MA (see section Electron-stimulated desorption from condensed Fe(CO)_4_MA), start to emerge only above 1 mTorr (Figure S7, Supporting Information File 1). This is similar to the behavior observed for Fe(CO)_5_ [27] and points again to dissociative adsorption of the monolayer that is formed below 1 mTorr of vapor, after which intact precursor condenses on the decomposed monolayer.

### Electron irradiation and deposition experiments

Volatile neutral molecules or fragments thereof that desorbed during electron irradiation of precursor layers with a mean thickness of roughly five monolayers and held at 100 K were monitored using the QMS in an ESD experiment. This was either done in a mass scan mode or as QMS signal versus time mode for selected *m*/*z* ratios. In general, electron irradiation was performed at an energy of 50 eV, but one particular experiment at 100 eV is included for comparison. Each ESD experiment was followed by a post-irradiation TDS run to record volatile species that desorb thermally upon increase of the substrate temperature.

Three different processes for deposition of iron from the precursors were performed. Each individual deposition experiment took about one day to perform. The gas doses applied in each individual step of the experiments corresponded to a pressure drop of 5 mTorr in the gas inlet system. Leakage of such doses was typically performed within 3 min. The EBID process was simulated by dosing the precursor vapor onto the Ta substrate at room temperature during electron exposure with an energy of 50 eV and a total electron dose of 10000 µC/cm^2^ at a typical current density of 15–17 µA/cm^2^. The irradiation time of roughly 10 min was sufficient to allow the chamber pressure to return to a value that was roughly the same as before the precursor leakage. After irradiation, a TDS run was performed to remove remaining volatile compounds. To perform a cryo-EBID process, the precursor was leaked onto the Ta substrate held at 100 K. The same electron exposure as used for EBID was then applied to the condensed precursor layer, followed by TDS. Finally, AG experiments were performed by preparing an EBID deposit from Fe(CO)_5_ as described above and leaking either Fe(CO)_5_ or Fe(CO)_4_MA onto the deposit at room temperature without electron irradiation. For each AG step, this was again followed by TDS. Each deposition experiment was performed twice, which revealed, in general, that the results were reproducible. A third repetition was not performed because the aim was to do all experiments within a relatively short time span to avoid effects of degradation of the precursor.

### Deposit characterization

AES data were recorded at an incident energy of 5 keV with pulse counting in the fixed retarding ratio (FRR) mode with a variable energy resolution of d*E*/*E* = 0.6%. 100 energy scans from 100 to 800 eV were accumulated in about 60 min with a beam current measured on the deposits around 0.3 µA and a beam spot size of roughly 1 mm diameter. To avoid accumulated surface damage from the electron beam, spectra were recorded at different positions of the substrate in each step of sequential experiments. AES acquisitions were generally performed with the substrate held at room temperature. Differential AES data with respect to the electron energy were obtained numerically after baseline correction using the asymmetric least squares method and were smoothed using a Savitzky–Golay filter. The deposit growth was characterized by monitoring the increase of the peak-to-peak heights in the differential AES data with each deposition step. The composition of the deposits was calculated by correcting the peak-to-peak heights with the corresponding tabulated sensitivity factors for Fe_LMM_ (0.9168 at 705 eV), C_KLL_ (0.4763 at 275 eV), and O_KLL_ (1.1012 at 510 eV) [54].

Deposit growth was also monitored based on the attenuation of the Ta_NNN_ signal of the sputter-cleaned Ta substrate with each process step following standard procedures [54]. Because the deposits contain more than one element, a lower and upper limit of the thickness that a deposit with homogeneous thickness would have was calculated using the electron attenuation length (EAL) of Ta_NNN_ Auger electrons in Fe (0.38 nm at 183 eV) as well as in C (0.64 nm at 183 eV). The attenuation lengths were derived by interpolation of tabulated values from [55]. The EAL of C_KLL_, O_KLL_, and Fe_LMM_ Auger electrons in Fe (0.48 nm at 275 eV, 0.74 eV at 510 eV, and 0.96 nm at 705 eV) and C (0.87 nm at 275 eV, 1.41 nm at 510 eV, and 1.86 nm at 705 eV) were used to discuss the evolution of the signal intensities in the two deposition steps of each experiment.

### Calculations

The Gibbs free energies for loss of MA or one CO ligand from Fe(CO)_4_MA were calculated in Dallas using the widely used hybrid functionals B3LYP and B97D, which include a dispersion correction, with the LANL2DZ basis set for Fe and the 6-311+G(2d,p) basis set for the other elements using Gaussian 09 [66]. The complex and the resulting fragments were geometry optimized. The resulting structure of Fe(CO)_4_MA was consistent with previously reported data [63,67].

## Supporting Information

Supporting Information features ESD (Figures S1 and S2) and post-irradiation TDS (Figure S3) data for Fe(CO)_5_, ESD at 20 eV electron energy (Figure S4) and corresponding post-irradiation TDS (Figure S5) for Fe(CO)_4_MA, a summary of AES intensities and data calculated from those for Fe(CO)_5_ (Table S1) and Fe(CO)_4_MA (Table S2), a plot of EAL values as function of electron energy and attenuation as function of overlayer thickness (Figure S6), TDS data for Fe(CO)_4_MA as function of coverage (Figure S7), and ^1^H NMR (Figure S8) and IR (Figure S9) spectra of Fe(CO)_4_MA.

File 1 Additional figures and tables.

## Data Availability

The data that supports the findings of this study is available from the corresponding author upon reasonable request.
