# Supplementary material for: Electron-induced deposition using Fe(CO)4MA and Fe(CO)5 – effect of MA ligand and process conditions
Source: Beilstein J Nanotechnol. 2024 May 8;15:500–16. doi: 10.3762/bjnano.15.45 (PMC11092064; doi:10.3762/bjnano.15.45)
Supplement: File 1 — Additional figures and tables. [file Beilstein_J_Nanotechnol-15-500-s001.pdf]

BEILSTEIN JOURNAL OF NANOTECHNOLOGY

Supporting Information

for

Electron-induced deposition using $Fe(CO)_4$MA and $Fe(CO)_5$ – effect of MA ligand and process conditions

Hannah Boeckers, Atul Chaudhary, Petra Martinović, Amy V. Walker, Lisa McElwee-White and Petra Swiderek

Beilstein J. Nanotechnol. **2024,** *15,* 500–516. doi:10.3762/bjnano.15.45

Additional figures and tables

License and Terms: This is a supporting information file under the terms of the Creative Commons Attribution License (https://creativecommons.org/licenses/by/4.0). Please note that the reuse, redistribution and reproduction in particular requires that the author(s) and source are credited and that individual graphics may be subject to special legal provisions.
The license is subject to the *Beilstein Journal of Nanotechnology* terms and conditions: (https://www.beilstein-journals.org/bjnano/terms)

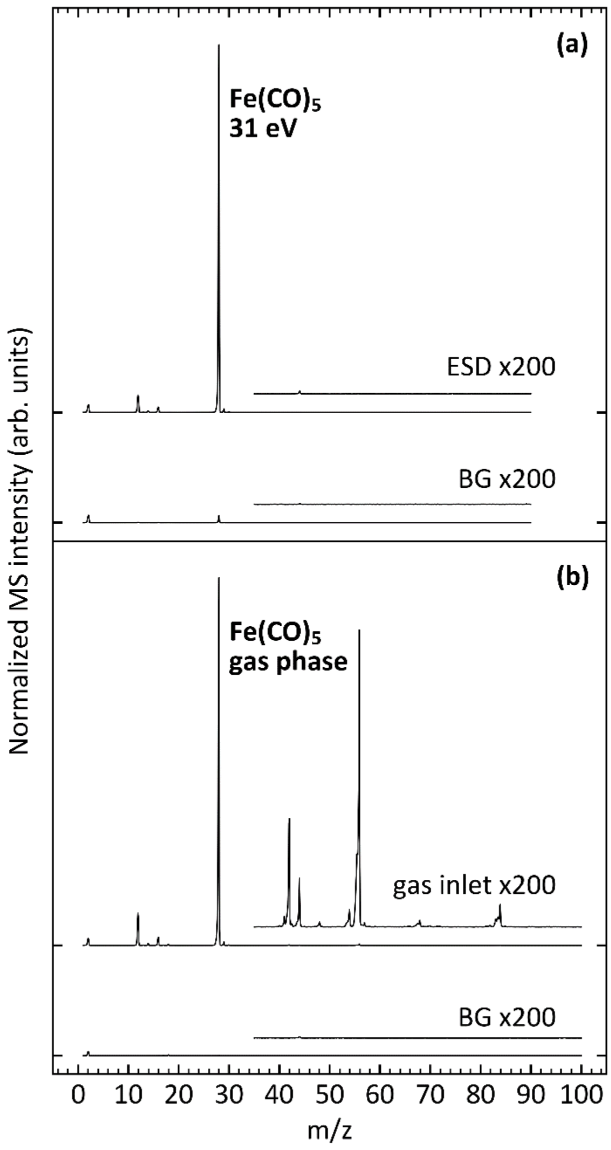

Figure S1: (a) ESD of neutral species during irradiation with 31 eV electrons from an $Fe(CO)_5$ multilayer on a Ta substrate held at 100 K (ESD). The thickness of the condensed layer was roughly 4 monolayers. (b) Mass spectra recorded during leaking of $Fe(CO)_5$ into the UHV chamber (gas inlet). Both data sets include a background mass spectrum (BG) recorded immediately prior to precursor leakage and electron irradiation, respectively. Ticks on the vertical axis indicate the baseline for each curve.

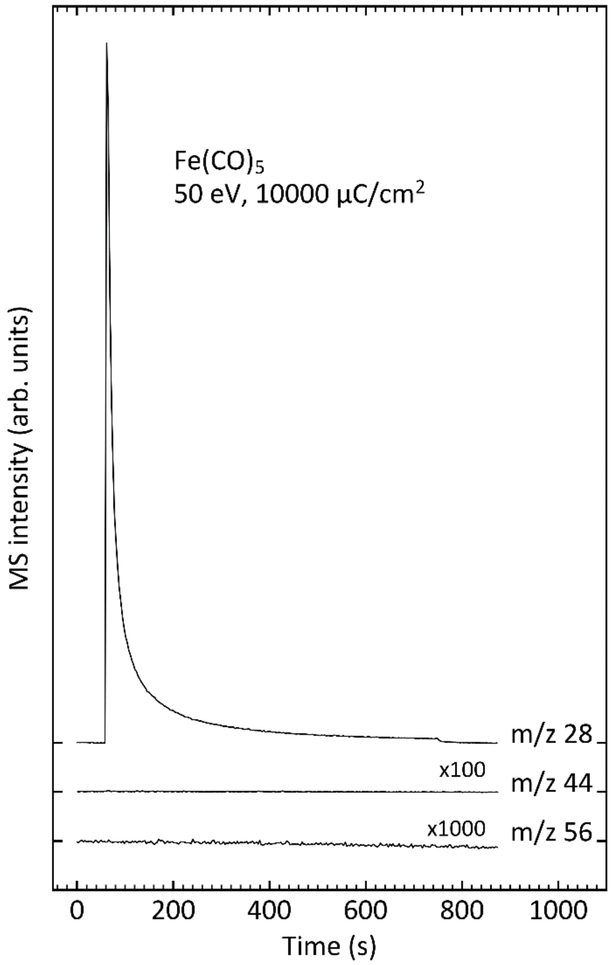

Figure S2: Time evolution of characteristic ESD signals recorded during irradiation with 50 eV electrons from an $Fe(CO)_5$ multilayer on a Ta substrate held at 100 K. The thickness of the condensed layer was roughly 5 monolayers. *m/z* 28 represents desorption of CO, *m/z* 56 was recorded to determine if Fe-containing species desorb. *m/z* 44 was monitored to search for desorption of CO_2. The total electron exposure was 10000 $\mu C/cm^2$. Ticks on the vertical axis indicate the baseline for each curve at the beginning of the experiment.

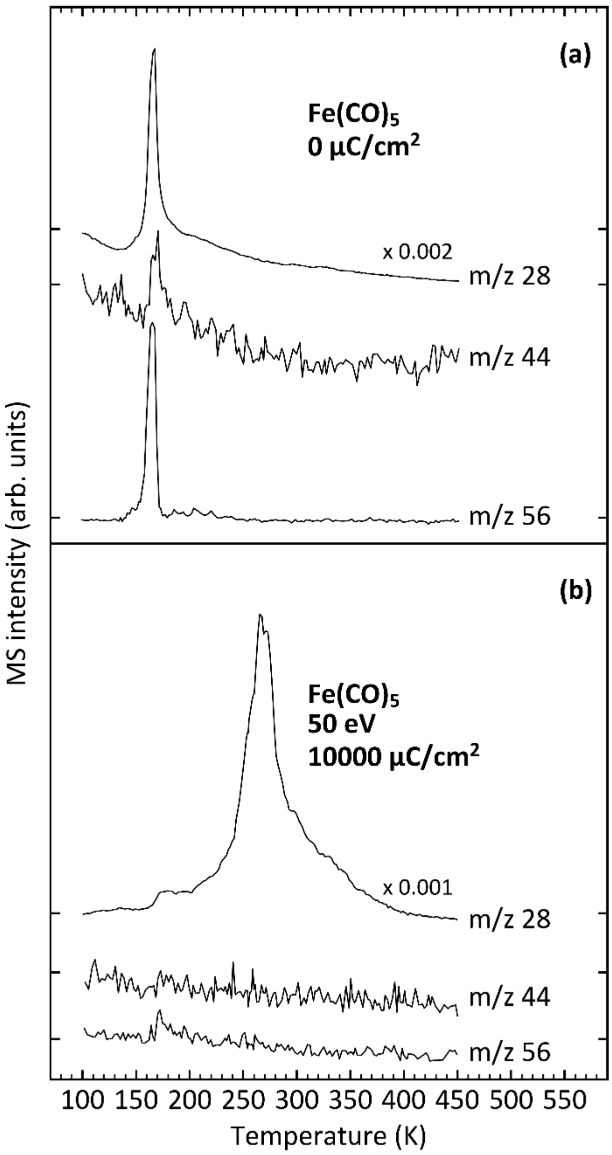

Figure S3: TDS obtained (a) from a pristine $Fe(CO)_5$ multilayer and (b) from an $Fe(CO)_5$ multilayer after irradiation with a total electron exposure of 10000 μC/cm^2 at 50 eV shown in Figure S2. The thickness of the condensed layers prepared on a Ta substrate held at 100 K was roughly 5 monolayers. Ticks on the vertical axis indicate the baseline for each curve at the beginning of the experiment. A slope in the baseline was due to pumping of residual precursor gas from the chamber.

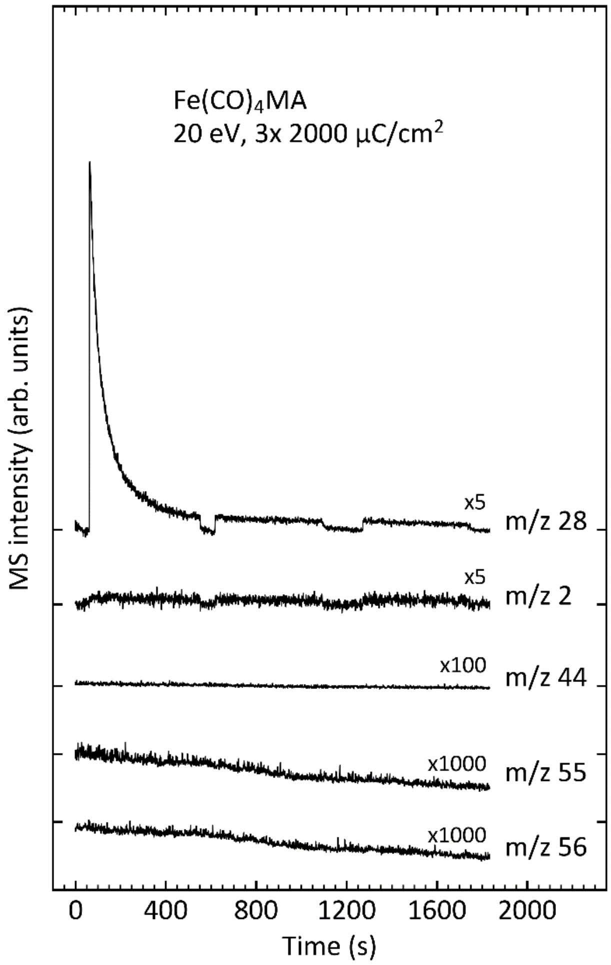

Figure S4: Time evolution of characteristic ESD signals from an $Fe(CO)_4MA$ multilayer on a Ta substrate held at 100 K, recorded during irradiation with 20 eV electrons. The thickness of the condensed layer was roughly 5 monolayers. *m/z* 28 represents desorption of CO, *m/z* 2 is H_2, and *m/z* 55 and 56 were recorded to determine if Fe-containing species or the free ligand or fragments thereof desorb. *m/z* 44 was monitored to search for desorption of CO_2. Irradiation was performed in three steps, each consisting of electron exposures of 2000 $\mu C/cm^2$. Ticks on the vertical axis indicate the baseline for each curve at the beginning of the experiment. A slope in the baseline was due to pumping of residual precursor gas from the chamber.

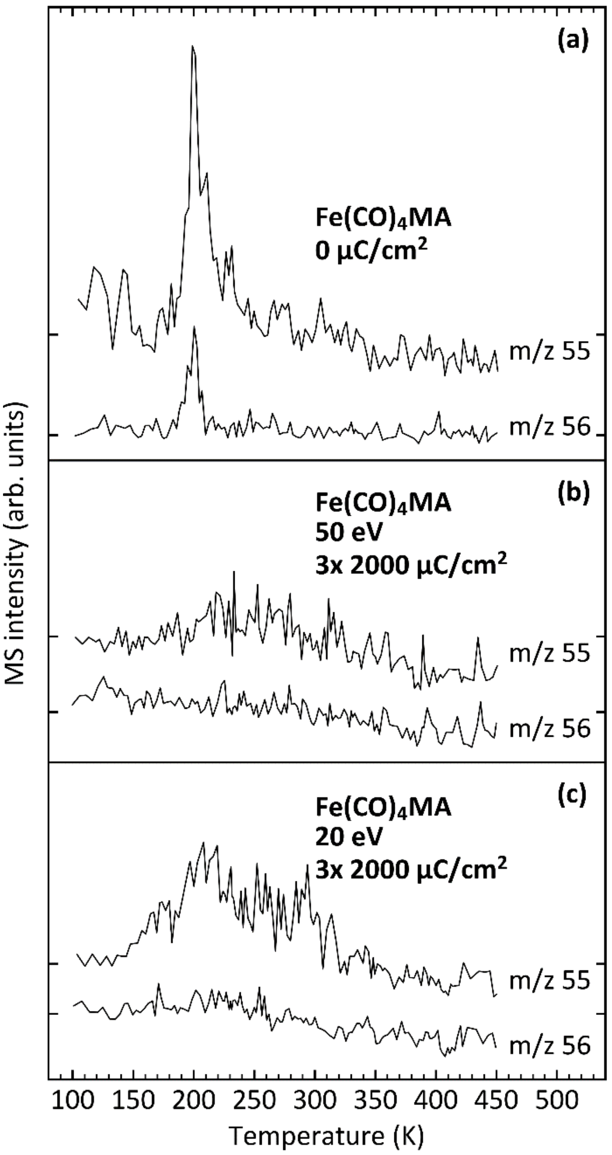

Figure S5: TDS obtained (a) from a pristine $Fe(CO)_4MA$ multilayer without electron irradiation (denoted as 0 μC/cm^2) and (b) from $Fe(CO)_4MA$ multilayers after three irradiation steps with a total electron exposure of 6000 μC/cm^2 at 50 eV, and (c) after the same irradiation experiment but performed at 20 eV. The thickness of the condensed layers prepared on a Ta substrate held at 100 K was roughly 5 monolayers. Ticks on the vertical axis indicate the baseline for each curve at the beginning of the experiment. A slope in the baseline was due to pumping of residual precursor gas from the chamber.

Table S1: Summary of deposition experiments by thermal reactions (TDS), room temperature EBID, cryo-EBID, and autocatalytic growth (AG) using $Fe(CO)_5$.

**$Fe(CO)_5$**	AES PtP heights				Thickness [nm][a]	Fe : C : O (at%)[b]
	Ta	C	O	Fe		
TDS experiments (precursor condensed at 100 K followed by warm-up):<br>Sputtering / AES / 2x TDS (2.5 mTorr) / AES / 2x TDS (2.5 mTorr) / AES						
TDS w/o precursor	62372<br>62595<br>61545	4438<br>7225<br>8678	6969<br>19228<br>32633	0<br>0<br>0	 0 /0 (1)<br>0.01/0.01 (2)	 0 : 1 : 1.2<br>0 : 1 : 1.6
TDS 1	55059<br>36478<br>31384	9181<br>25619<br>28628	11204<br>43528<br>47306	0<br>5516<br>7702	 0.16/0.26 (2x)<br>0.21/0.36 (4x)	 1 : 8.9 : 6.6<br>1 : 7.2 : 5.1
TDS 2	55874<br>38256<br>32856	9978<br>27793<br>29579	10426<br>44674<br>46932	0<br>5313<br>6828	 0.14/0.24 (2x)<br>0.20/0.34 (4x)	 1 : 10.1 : 7.0<br>1 : 8.3 : 5.7
EBID experiment (precursor dosing at room temperature during electron irradiation):<br>Sputtering / AES / EBID (5 mTorr) / AES / EBID (5 mTorr) / AES						
EBID w/o precursor<br>50 eV	62396<br>62684<br>56943	4049<br>10064<br>11873	6098<br>27564<br>45537	0<br>0<br>0	 0 /0 (1)<br>0.04/0.06 (2)	 0 : 1 : 1.2<br>0 : 1 : 1.7
EBID 1, 50 eV	57483<br>37552<br>29259	8904<br>22304<br>20180	15985<br>54787<br>66745	0<br>9628<br>20019	 0.16/0.27 (1)<br>0.26/0.43 (2)	 1 : 4.5 : 4.7<br>1 : 1.9 : 2.8
EBID 2, 50 eV	57567<br>36336<br>24071	8698<br>22804<br>18468	13721<br>55371<br>60976	0<br>12694<br>24382	 0.17/0.29 (1)<br>0.33/0.56 (2)	 1 : 3.5 : 3.6<br>1 : 1.5 : 2.1
Cryo-EBID experiments (precursor condensed at 100 K, then electron irradiation and warm-up):<br>Sputtering / AES / cryo-EBID (5 mTorr) / AES / cryo-EBID (5 mTorr) / AES						
Cryo-EBID 1, 50 eV	57543<br>23787<br>13511	7865<br>24736<br>25533	10700<br>43934<br>42896	0<br>28178<br>44919	 0.34/0.57 (1)<br>0.55/0.93 (2)	 1 : 1.7 : 1.3<br>1 : 1.1 : 0.8
Cryo-EBID 2, 50 eV	56061<br>23060<br>13543	7609<br>23849<br>23055	10971<br>41354<br>42903	0<br>28883<br>45878	 0.34/0.57 (1)<br>0.54/0.91 (2)	 1 : 1.6 : 1.2<br>1 : 1.0 : 0.8
Autocatalytic growth experiments (EBID seed deposit from $Fe(CO)_5$, then thermal growth):<br>Sputtering / AES / EBID (5 mTorr $Fe(CO)_5$) / AES / AG (5 mTorr) / AES / AG (5 mTorr) / AES						
AG 1	65309<br>41283<br>31209<br>22389	4714<br>29583<br>26257<br>22615	6953<br>49171<br>56175<br>57624	0<br>12860<br>26249<br>40104	 0.17/0.29 (EBID)<br>0.28/0.47 (1)<br>0.41/0.69 (2)	 1 : 4.4 : 3.2<br>1 : 1.9 : 1.8<br>1 : 1.1 : 1.2
AG 2	66904<br>38819<br>29736<br>21583	5003<br>27646<br>24833<br>21907	6877<br>50353<br>54703<br>56148	0<br>13302<br>23929<br>35208	 0.21/0.35 (EBID)<br>0.31/0.52 (1)<br>0.43/0.72 (2)	 1 : 4.0 : 3.2<br>1 : 2.0 : 1.9<br>1 : 1.2 : 1.3

[a] Thickness calculated from the attenuation of the Ta signal. Upper and lower values result from electron attenuation lengths of Ta_{NNN} electrons in Fe (0.38 nm at 183 eV) as well as in C (0.64 nm at 183 eV) [1], respectively.

[b] Composition derived from peak-to-peak heights of the AES signals weighted by tabulated sensitivity factors at 5 keV for the elements Fe (0.9168 at 705 eV), C (0.4763 at 275 eV), and O (1.1012 at 510 eV) [2] assuming a homogeneous distribution of the elements within the deposit.

Table S2: Summary of deposition experiments by thermal reactions (TDS), room temperature EBID, cryo-EBID, and autocatalytic growth (AG) using $Fe(CO)_4MA$.

**$Fe(CO)_4MA$**	AES PtP heights				Thickness [nm][a]	Fe : C : O (at%)[b]
	Ta	C	O	Fe		
TDS experiments (precursor condensed at 100 K followed by warm-up):<br>Sputtering / AES / 2x TDS (2.5 mTorr) / AES / 2x TDS (2.5 mTorr) / AES						
TDS w/o precursor	62372<br>62595<br>61545	4438<br>7225<br>8678	6969<br>19228<br>32633	0<br>0<br>0	 0 /0 (1)<br>0.01/0.01 (2)	 0 : 1 : 1.2<br>0 : 1 : 1.6
TDS 1	57035<br>35532<br>28704	7017<br>27409<br>29780	12060<br>46533<br>49356	0<br>5650<br>5998	 0.18/0.30 (2x)<br>0.26/0.44 (4x)	 1 : 9.3 : 6.9<br>1 : 9.5 : 6.9
TDS 2	55494<br>33760<br>29460	8934<br>28382<br>30940	11226<br>41311<br>46577	0<br>5134<br>7115	 0.19/0.32 (2x)<br>0.24/0.41 (4x)	 1 : 10.6 : 6.7<br>1 : 8.4 : 5.5
EBID experiment (precursor dosing at room temperature during electron irradiation):<br>Sputtering / AES / EBID (5 mTorr) / AES / EBID (5 mTorr) / AES /						
EBID w/o precursor<br>50 eV	62396<br>62684<br>56943	4049<br>10064<br>11873	6098<br>27564<br>45537	0<br>0<br>0	 0.00/0.00 (1)<br>0.04/0.06 (2)	 0 : 1 : 1.2<br>0 : 1 : 1.7
EBID 1, 50 eV	57299<br>32783<br>18405	15401<br>28900<br>29867	21606<br>57368<br>65725	0<br>11517<br>23188	 0.21/0.36 (1)<br>0.43/0.73 (2)	 1 : 4.7 : 3.7<br>1 : 2.5 : 2.4
EBID 2, 50 eV	57543<br>30679<br>15802	9569<br>29458<br>29511	16819<br>53790<br>68985	0<br>12021<br>24785	 0.24/0.40 (1)<br>0.49/0.83 (2)	 1 : 4.8 : 4.1<br>1 : 2.3 : 2.3
EBID 1, 100 eV	59037<br>26394<br>12250	6984<br>30956<br>31999	13706<br>51263<br>51421	0<br>15949<br>34499	 0.31/0.51 (1)<br>0.60/1.00 (2)	 1 : 3.7 : 2.7<br>1 : 1.8 : 1.2
EBID 2, 100 eV	53708<br>23174<br>12211	7202<br>30853<br>30763	8644<br>43860<br>42828	0<br>18021<br>31398	 0.32/0.54 (1)<br>0.56/0.95 (2)	 1 : 3.3 : 2.0<br>1 : 1.9 : 1.1
Cryo-EBID experiments (precursor condensed at 100 K, then electron irradiation and warm-up):<br>Sputtering / AES / cryo-EBID (5 mTorr) / AES / cryo-EBID (5 mTorr) / AES						
Cryo-EBID 1, 50 eV	55504<br>8266<br>2697	6975<br>27479<br>28042	10461<br>45190<br>44556	0<br>19930<br>23647	 0.72/1.22 (1)<br>1.15/1.94 (2)	 1 : 2.7 : 1.9<br>1 : 2.3 : 1.6
Cryo-EBID 2, 50 eV	54222<br>6792<br>2827	6949<br>27112<br>29151	9343<br>43469<br>46850	0<br>19126<br>24854	 0.79/1.33 (1)<br>1.12/1.90 (2)	 1 : 2.7 : 1.9<br>1 : 2.3 : 1.6
Autocatalytic growth experiments (EBID seed deposit from $Fe(CO)_5$, then thermal growth):<br>Sputtering / AES / EBID (5 mTorr $Fe(CO)_5$) / AES / AG (5 mTorr) / AES / AG (5 mTorr) / AES						
AG 1	55154<br>34316<br>27555<br>23593	5525<br>24225<br>26168<br>28045	8412<br>45809<br>48466<br>52627	0<br>11581<br>13479<br>15689	 0.18/0.30 (EBID)<br>0.26/0.44 (1)<br>0.32/0.54 (2)	 1 : 4.0 : 3.3<br>1 : 3.7 : 3.0<br>1 : 1.8 : 2.8
AG 2	55527<br>34241<br>33530<br>27608	5486<br>24407<br>34105<br>34600	6489<br>44110<br>57444<br>58881	0<br>11555<br>17238<br>20967	 0.18/0.31 (EBID)<br>0.19/0.32 (1)<br>0.27/0.45 (2)	 1 : 4.0 : 3.2<br>1 : 3.8 : 2.8<br>1 : 1.7 : 2.8

[a] Thickness calculated from the attenuation of the Ta signal. Upper and lower values result from electron attenuation lengths of Ta_{NNN} electrons in Fe (0.38 nm at 183 eV) as well as in C (0.64 nm at 183 eV) [1], respectively.
[b] Composition derived from peak-to-peak heights of the AES signals weighted by tabulated sensitivity factors at 5 keV for the elements Fe (0.9168 at 705 eV), C (0.4763 at 275 eV), and O (1.1012 at 510 eV) [2] assuming a homogeneous distribution of the elements within the deposit.

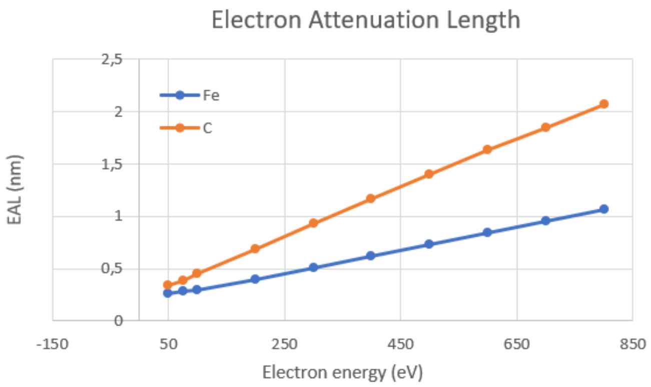

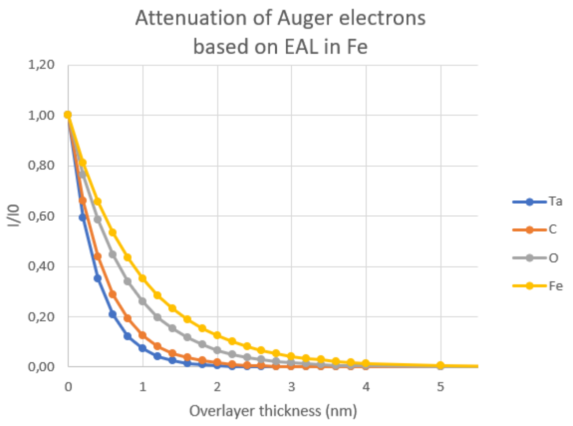

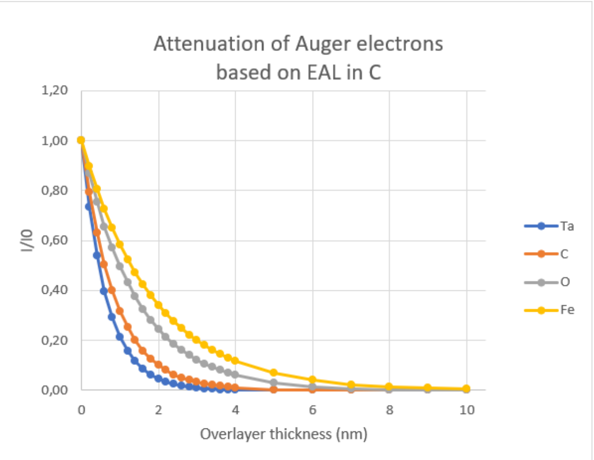

Figure S6: (Top) Electron attenuation length as function of electron energy in pure Fe and C, respectively [1]. These values are used to obtain a lower and upper limit of the deposit thickness from the attenuation of the Ta_{NNN} AES signal as summarized in Tables S1 and S2. (Bottom) Attenuation of Ta_{NNN}, C_{KLL}, O_{KLL}, and Fe_{LMM} AES signal intensities originating from below an overlayer with varying thickness and consisting of pure Fe (left) and C (right), respectively. The intensities were obtained as $I = I_0 \cdot e^{-d/EAL}$ with overlayer thickness d and electron attenuation length EAL for Fe and C, respectively.

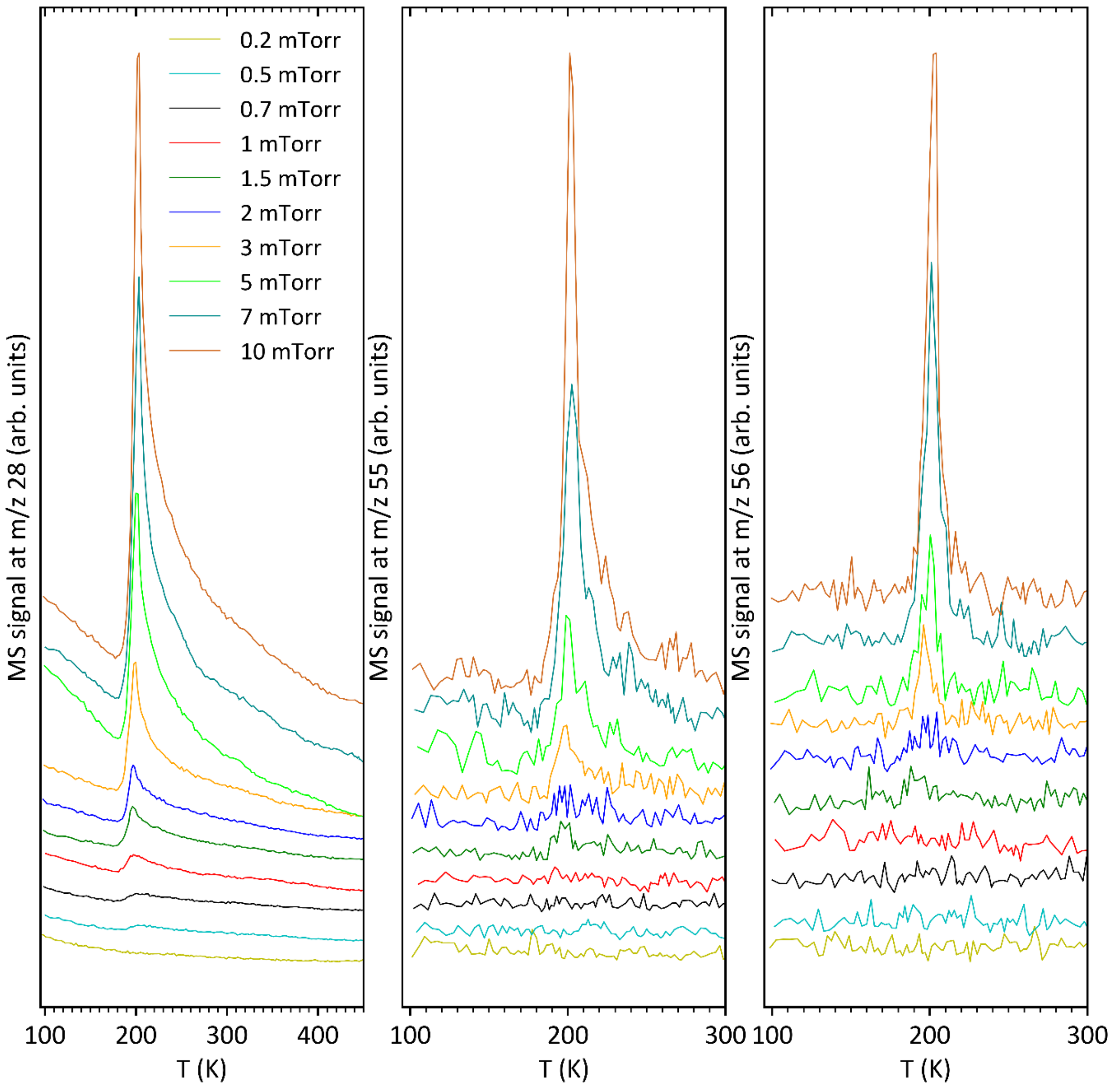

Figure S7: TDS curves obtained for *m/z* 28 (left), *m/z* 55 (middle), and *m/z* 56 (right) after leaking varying amounts of $Fe(CO)_4MA$ onto the Ta substrate. The gas dose is defined as the pressure drop in the gas handing manifold in units of mTorr. A desorption signal in the *m/z* 28 TDS curves ($CO^{\bullet+}$) already appears well below a gas dose of 1 mTorr and develops into a sharp peak near 200 K above that dose. In contrast, signals in the *m/z* 55 and 56 curves ($CH_2CHCO^{\bullet+}$, the dominant fragment of MA, and Fe^+, respectively), indicative of desorption of $Fe(CO)_4MA$, start to emerge only above 1 mTorr. The slope of the baseline that is most noticeable in the *m/z* 28 TDS curves is due to pumping of residual gas in the UHV chamber after leaking of the precursor.

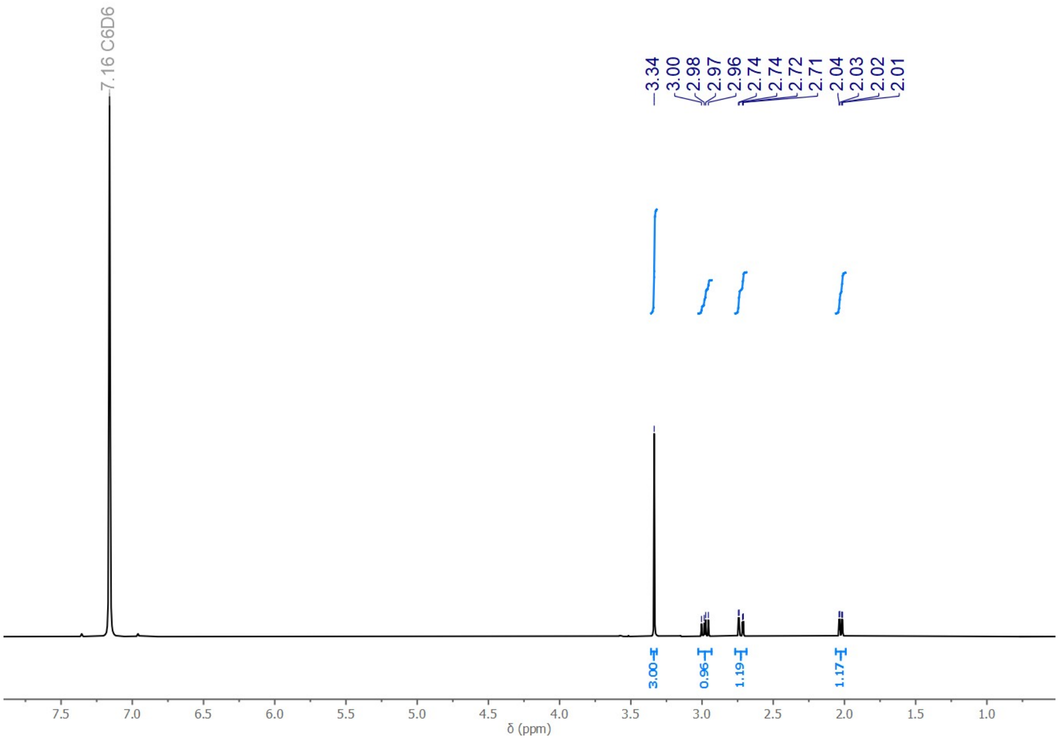

Figure S8: ^{1}H NMR (400 MHz, 25 °C, C_6D_6) spectrum of $Fe(CO)_4$MA (MA = methyl acrylate, H_2C=CH-$COOCH_3$): δ 3.34 (s, 3H), 2.98 (dd, J = 11.6, 7.8 Hz, 1H), 2.73 (dd, J = 11.6, 2.3 Hz, 1H), 2.03 (dd, J = 7.7, 2.3 Hz, 1H).

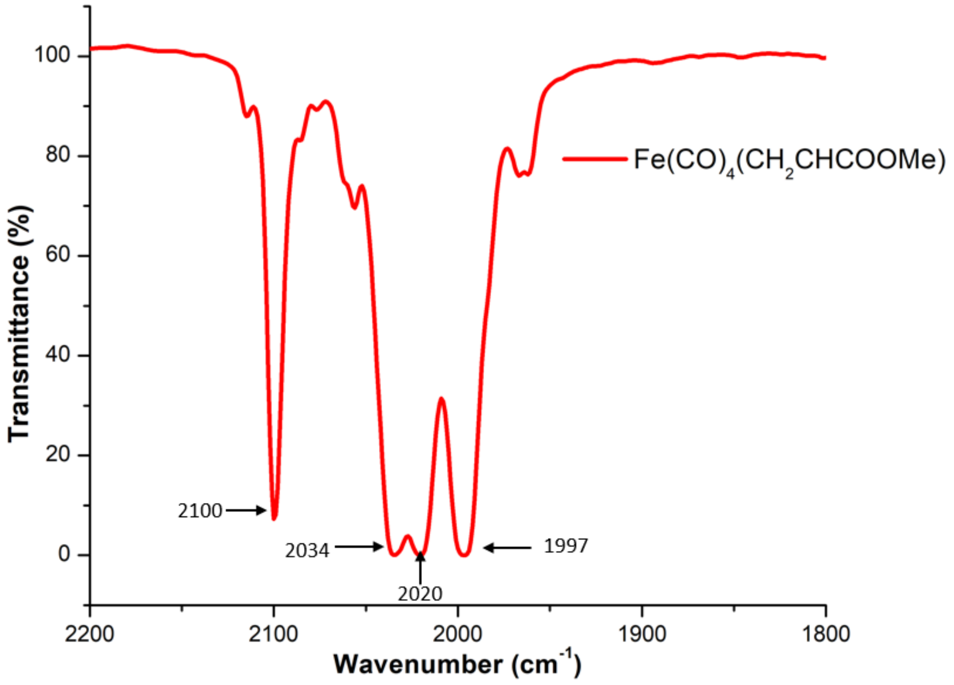

Figure S9: FT-IR spectrum of $Fe(CO)_4$MA (MA = methyl acrylate, H_2C=CH-$COOCH_3$) obtained in hexane solution: ν_{CO} (cm^{-1}): 2100, 2034, 2020, 1997.

References

[1] P. J. Cumpson and M. P. Seah, Surf. Interface Anal. **25,** 430 (1997).

[2] D. Briggs and J. T. Grant (eds), Surface analysis by Auger and X-ray photoelectron spectroscopy (IM Publications, Chichester, UK and Surface Spectra, Manchester, UK, 2003).
